# Respiratory Sinus Arrhythmia—Common and Distinct Mechanisms of Emotional Adjustment in the Depressive and Anxiety Disorders Spectrum?

**DOI:** 10.1111/psyp.70079

**Published:** 2025-05-31

**Authors:** Dirk Adolph, Xiao Chi Zhang, Tobias Teismann, Andre Wannemüller, Jürgen Margraf

**Affiliations:** ^1^ Mental Health Research and Treatment Center Ruhr University Bochum Bochum Germany

**Keywords:** anxiety, depression, prediction of treatment outcome, respiratory sinus arrhythmia, transdiagnostic approach

## Abstract

Respiratory sinus arrhythmia (RSA) reflects the activity of a cortico‐limbic control system, enabling the flexible regulation of cardiac output via the parasympathetic nervous system. We assessed two markers of RSA, that is resting RSA (rRSA) and RSA reactivity (ΔRSA) and evaluated their common and distinct role for regulating emotional reactivity across depressive and anxiety disorders and their treatments. We recruited samples of healthy controls and patients with anxiety and depressive disorders, assessed rRSA during baseline and ΔRSA as RSA change from baseline to viewing emotional films. Patients then underwent disorder‐specific cognitive behavior therapy. Although both patient groups exhibited lower rRSA than controls, depression—but not anxiety—symptomatology was transdiagnostically associated with less rRSA and ΔRSA. Complementing these depression‐specific results, better ΔRSA predicted better treatment outcome in depression, but not anxiety. Our data confirm RSA as a transdiagnostic marker for mood and anxiety, support recent attempts toward transdiagnostic, dimensional classification systems (HiToP, RDoC) and provide evidence for a more robust association of RSA with depression symptomatology and treatment. This renders rRSA and ΔRSA potential markers to assess common *and* distinct mechanisms associated with depression and anxiety.

## Abstract 2

On the one hand, our data confirm previous findings on a relationship between low *resting RSA* and depression and anxiety. On the other hand, our data provide compelling new evidence for a more robust association of flattened *RSA reactivity* with depression symptomatology and depression treatment as compared to anxiety. Our study is thus the first to demonstrate that different indices of RSA (resting RSA, RSA reactivity) constitute potential biomarkers to assess common *and* distinct emotion‐related mechanisms underlying depression and anxiety and their treatments.

## 1 Introduction

Although current nosologic systems list depressive and anxiety disorders as distinct disorder categories, they are highly comorbid (Gorman 1996; Kessler et al. 2005; Sartorius et al. 1996) and share a considerable amount of symptomatology (Clark and Watson 1991). Consequently, there is still debate on how these disorders are separable in terms of their underlying pathogenic processes (Mineka et al. 1998). Indeed, while depression and anxiety share a liability toward enhanced negative affectivity (Shankman and Klein 2003), there is also consensus between current models that many anxiety disorders (e.g., panic disorder, specific phobias or PTSD), but not depressive disorders, are related to elevated threat responses and physiological hyperarousal (Dillon et al. 2014; Joiner et al. 1999; McTeague and Lang 2012; Shankman and Klein 2003). In turn, with some exceptions, low positive affect (Shankman and Klein 2003) or blunted emotional reactivity toward a broad range of emotional stimuli (cf. emotion context insensitivity hypothesis of depression, Bylsma 2021; Rottenberg, Gross, et al. 2005) is related to depressive rather than anxiety disorders. Several lines of research so far have focused on identifying objective markers to disentangle these common and distinct emotion‐related mechanisms underlying both disorders (Beauchaine et al. 2019; Chesnut et al. 2021; Lacerda‐Pinheiro et al. 2014; Rafter 2001; de Zorzi et al. 2021). Although previous research has argued that within this endeavor, Respiratory Sinus Arrhythmia (RSA) might be a promising candidate (Beauchaine 2015; Beauchaine and Thayer 2015), to the best of our knowledge, no study to date assessing RSA has included patients from the depressive and anxiety disorders spectrum at the same time in one study. The current research aimed at closing this gap. We assessed two common markers of RSA, that is resting RSA (rRSA) and RSA reactivity (ΔRSA), recruited a naturalistic sample of treatment‐seeking patients with depressive and anxiety disorders as well as healthy controls, and analyzed the significance these markers have within and across the depressive and anxiety disorders as well as the predictive validity both markers have for the outcome of cognitive behavior therapy (CBT).

### 1.1 RSA and Psychopathology

RSA is an index of cardiac parasympathetic control and reflects the activity of a cortico‐limbic control system, enabling the flexible regulation of cardiac output via the vagus nerve and the parasympathetic nervous system. RSA is characterized by the variation in heart rate during the breathing cycle. During inhalation, the activity of the vagus nerve is reduced, leading to an increase in heart rate, whereas during exhalation, vagus nerve activity increases, resulting in a decrease in heart rate (overview in Quigley et al. 2024). Thereby, high levels of rRSA have been previously associated with indices of self‐regulation and cognitive control, whereas low rRSA has been associated with measures of cognitive inflexibility (Beauchaine 2015; Laborde et al. 2023). In contrast to rRSA, ΔRSA reflects the momentary change in RSA levels from a resting state to an emotional (either positive or negative) or stress‐related situation. That is, while at rest (e.g., while sitting in a relaxing position), the parasympathetic nervous system acts as a brake, slowing heart rate and promoting homeostasis (Porges 1995; Thayer and Lane 2000; Quigley et al. 2024). When facing significant emotional situations (like for example while performing a stressful cognitive, emotional or physical task), this “vagal brake” is released (i.e., vagal withdrawal), initiating a decrease in parasympathetic activity and an increase in heart rate to facilitate—in concert with the sympathetic nervous system—an adaptive physiological response to situational demands (Campbell and Wisco 2021; Porges 1995; Thayer and Lane 2000). Thus, rRSA (as for example measured during a resting baseline) can roughly be seen as a marker for the organism's general potential for adaptive physiological reactivity to emotional or stressful challenges, while ΔRSA (measured for example as the change in RSA from a resting baseline to a specific demanding task or situation) reflect the organism's efficiency in recruiting these reserves in the light of environmental demands (Hamilton and Alloy 2016; Smith et al. 2020). It has been suggested that this healthy adaptation system is impaired in psychopathology, including depression and anxiety (Beauchaine 2015), and that both rRSA and ΔRSA might contribute uniquely to between‐subject variance in psychopathology (Yaroslavsky et al. 2013).

However, despite these theoretical considerations, the literature on the unique role these parasympathetic indices play to differentiate depressive and anxiety disorders is contradictory. In detail, confirming general deficits in emotion adaptation and in line with current theoretical models (Shankman and Klein 2003), meta‐analytical work has shown that depressive and anxiety disorders are associated with low levels of rRSA (Chalmers et al. 2014; Koch et al. 2019). In contrast, while cumulated evidence suggests blunted ΔRSA in depression (Hamilton and Alloy 2016; Schiweck et al. 2019) a recent review did not provide a clear picture of whether anxiety is associated with deviant ΔRSA (Campbell and Wisco 2021). Likewise, while there is initial evidence suggesting predictive validity of both rRSA and ΔRSA for depression treatment (Blanck et al. 2019; Hage et al. 2017; Jain et al. 2014) there is only very limited research on the predictive validity of RSA for treatment outcome in anxiety disorders, yielding contradictory results (Lueken et al. 2016; Tolin et al. 2021). Taken together, although RSA has been conceptualized as a transdiagnostic biomarker for inflexible emotional reactivity in psychopathology (Beauchaine 2015; Beauchaine and Thayer 2015), research so far does not justify conclusive inferences about the differential effects these vagally mediated processes have within the anxiety and depressive disorders spectrum (Campbell and Wisco 2021; Hamilton and Alloy 2016; Schiweck et al. 2019).

### 1.2 The Current Study

Consequently, within the current study we aimed at closing this gap. We recruited a sample of patients with anxiety and depressive disorders, allowing for the evaluation of both unique and shared variance of RSA between anxiety and depression and across the entire anxiety and depressive disorders spectrum. We aimed to clarify whether there are interindividual differences in rRSA, as well as ΔRSA (i.e., RSA ractivity toward a range of emotional and neutral video clips) based on anxiety and depression psychopathology (cross‐sectional approach) and whether these two indices are relevant for the outcome of disorder‐specific cognitive behavioral treatments (longitudinal approach).

Both research questions were investigated concerning depression and anxiety as distinct diagnostic categories (using standardized diagnostic interviews to assess psychopthology) as well as in a transdiagnostic manner across disorder categories (using dimensional symptom measures, i.e., via questionnaires). The additional dimensional, transdiagnostic analysis of our data is compatible with the NIMH's Research Domain Criteria (RDoC) initiative (Cuthbert 2014). Traditional classification systems yield limited diagnostic reliability and often fail to account for comorbidity (Kessler et al. 2005) or symptom heterogeneity (Widiger and Trull 2007), probably as a direct consequence of categorizing dimensional phenomena (Forbes et al. 2021). Indeed, comorbidity within the anxiety and depression spectrum is present on the syndrome level and on the symptom level, and in clinical praxis, overlapping symptom patterns are the rule rather than the exception in patients with depressive and anxiety disorders. Given frequent syndromal and subsyndromal comorbid conditions, the intertwined relations between RSA deficits and anxiety/depression symptomatology can be sufficiently analyzed with a transdiagnostic approach only, assessing the symptom clusters of interest in a dimensional manner within a sample of participants showing a large range of the symptoms of interest (i.e., depression symptoms and anxiety symptoms in our case).

Thus, the additional transdiagnostic approach enabled us to consider the contribution of rRSA and ΔRSA as markers of affective self‐regulation and cognitive control within and across the anxiety and depressive disorders spectrum. Since we aimed for a naturalistic sample of patients attending for treatment at our outpatient center usually yielding high comorbidity rates between depression and anxiety, this approach is especially useful to disentangle the intertwined mechanisms underlying the depressive and anxiety disorders spectrum.

Within the current study, we control for a range of methodological issues identified in previous research (see Beauchaine et al. 2019). In brief, (1) we use negative film clips (i.e., fearful, sad), as well as a happy and neutral film to assess ΔRSA. This approach enables us to test if the ΔRSA response in depression, in direct comparison to anxiety, is based on global emotion context insensitivity; that is, the finding that depression is broadly associated with blunted emotional reactivity toward all emotional situations and stimuli (Bylsma 2021; Rottenberg, Gross, et al. 2005), which should lead to blunted ΔRSA toward *all* emotional film clips, or if reduced ΔRSA may be *restricted to disorder‐specific* films (e.g., the sad or happy film clip), which would speak in favor of specific emotion deficits rather than global emotion insensitivity (as suggested by previous theoretical models and corresponding data, for example, Rottenberg et al. 2002; Shankman and Klein 2003) (2) We strictly followed guidelines in RSA assessment and data reduction in terms of recommended sampling intervals and sampling rate (Berntson et al. 1997). (3) We controlled for medication, respiration, gender, and age. (4) We assessed the differential predictive validity that rRSA and ΔRSA have for treatment outcomes in depression and anxiety. We target RSA as a predictor for the outcome of CBT, mainly because it has been shown that CBT enhances global cognitive control network activity (Yang et al. 2018) as well as emotion regulation abilities (Forkmann et al. 2014), both of which processes have been previously associated with RSA (Beauchaine and Thayer 2015; Laborde et al. 2023). (5) To control for putative overlapping effects of the sympathetic nervous system, we additionally recorded the activity of the sympathetic branch of the autonomous nervous system (i.e., in terms of skin conductance level). We did so because it has been proposed that inconsistent findings in the literature so far may be founded at least in part on the consideration of isolated parts of the physiological regulation system (Fisher et al. 2021). Indeed, the two branches of the autonomous nervous system work in concert to promote homeostasis and to adapt the organism to current environmental demands, including stressful and/or emotional situations. Thus, to determine the specificity of the parasympathetic system, the concurrent assessment of sympathetic activity is advantageous.

Based on previous literature as outlined above, for our cross‐sectional approach, we hypothesize that both anxiety and depression are associated with reduced rRSA in comparison to healthy controls. Furthermore, if RSA is indeed transdiagnostically associated with psychopathology, a higher symptom load should be associated with lower rRSA. Concerning ΔRSA, following previous literature as outlined above, we hypothesize that ΔRSA in depression will be either reduced mainly toward the happy and sad film clips or following the emotion context insensitivity hypothesis toward the full range of emotion film clips. Based on previous findings, for our longitudinal approach, we hypothesize that markers of cardiac vagal control (i.e., higher rRSA and more pronounced ΔRSA) are predictive of treatment outcome for depression. Due to inconclusive previous literature, no predictions for the predictive validity of RSA markers for the outcome of CBT for anxiety disorders were possible.

## 2 Methods and Materials

### 2.1 Sample Characteristics

The current experiment was part of a larger study assessing mechanisms of anxiety disorders, depression, and their treatments carried out between 2013 and 2016. *N* = 223 patients from the Mental Health Research and Treatment Center of Bochum University participated. Of these, *n* = 27 did not qualify for a diagnosis of a depressive or an anxiety disorder and had to be excluded from the study. All participants gave written and informed consent to procedures. The study was conducted in accordance with the Declaration of Helsinki and was approved by the local ethics committee of the Faculty of Psychology at Ruhr University Bochum (Approval Number: Votum046). In addition to the patient sample, a total of *n* = 60 healthy adults (HC) agreed to participate in the current study. The patient sample comprised of depressed patients (DEP, total *n* = 103, *n* = 91 Major Depression, *n* = 9 Dysthymia/Persistend Depressive Disorder, *n* = 3 other depressive disorder) and patients with an anxiety disorder (ANX, total *n* = 93, *n* = 28 social phobia, *n* = 25 panic disorder/agoraphobia, *n* = 14 generalized anxiety disorder, *n* = 8 obsessive compulsive disorder, *n* = 8 post traumatic stress disorder, *n* = 6 specific phobia, *n* = 4 other anxiety disorder) attending for treatment at the outpatient clinic of the Mental Health Research and Treatment Center (MHRTC) at Ruhr University Bochum.^1^ A total of *n* = 22 patients with depression had a comorbid anxiety disorder, and a total of *n* = 31 patients with an anxiety disorder had a comorbid depressive disorder. All participants were Caucasian and recruited from the Ruhr Area in Germany. Diagnoses were obtained with standardized semi‐structured interviews for DSM‐IV disorders (Diagnostic Interview for Mental Disorders, DIPS, Schneider and Margraf 2006) by trained and certified postgraduate psychotherapists. The diagnoses obtained with the DIPS show very good interrater reliability (Kappa between *κ* = 0.72 and *κ* = 0.92; Suppiger et al. 2008; Schneider et al. 1992). Diagnoses of healthy controls were obtained with a brief semi‐structured interview for DSM‐IV disorders (MINI‐DIPS, Margraf 1994) and via self‐report. No control participant had to be excluded due to a current or history of mental disorders. A comprehensive sample description can be found in Table 1.

**TABLE 1:** Descriptive statistics of demographic variables and symptomatology for the three diagnostic groups.

	DEP	ANX	HC	Group comparisons
Age *M* (SD)	39.54 (14.68)	34.14 (11.91)	33.19 (11.29)	*F*(2, 249) = 6.04, *p* = 0.003^1,3^
Sex (m/f)	42/61	39/54	14/64	*χ* ^2^ (2) = 6.40, *p =* 0.041^1,2^
Psychotropic medication (yes/no)	56/47	33/60	0/60	*χ* ^2^ (2) = 49.45, *p* < 0.001^1,2,3^
DASS_General Distress_ *M* (SD)	28.33 (12.21)	23.67 (10.96)	9.32 (7.64)	*F*(2, 250) = 52.17, *p* < 0.001^1,2,3^
DASS_Depression_ *M* (SD)	11.34 (5.99)	7.20 (5.07)	2.32 (2.49)	*F*(2, 250) = 58.73, *p* < 0.001^1,2,3^
DASS_Anxiety_ *M* (SD)	5.62 (4.30)	6.46 (4.47)	1.66 (1.89)	*F*(2, 250) = 27.34, *p* < 0.001^1,2^
DASS_Stress_ *M* (SD)	11.47 (4.96)	10.59 (5.03)	5.34 (4.16)	*F*(2, 250) = 31.38, *p* < 0.001^1,2^

*Note:* Significant group comparisons (i.e., *p* < 0.05): 1 = HC versus DEP, 2 = HC versus ANX, 3 = DEP versus ANX.

Abbreviations: ANX, anxiety patients; DASS, depression anxiety and stress scale; DEP, depressed patients; HC, healthy controls.

### 2.2 Treatments

The patients received manual‐based disorder‐specific CBT as routinely carried out in our outpatient center (A list of manuals typically used in our center can be found in section 1 of the Supporting Information). The CBT treatments comprised approximately 25 sessions, and the number of sessions did not differ between depression and anxiety patients (DEP, *M* = 25.4, SD = 3.7; ANX, *M* = 25.9, SD = 2.6, *p* > 0.10). Treatments were carried out by therapists as part of their postgraduate training. All therapists had a masters' degree in Psychology and at least 1‐year full‐time postgraduate CBT training. They were additionally monitored by licensed CBT supervisors within regular supervision sessions (i.e., including discussions about the patient's current status and the ongoing treatment). However, despite general agreement with published manuals (e.g., exposure‐based CBT for anxiety disorders) treatments within routine outpatient care are usually less standardized than in typical randomized controlled trials (von Brachel et al. 2019). All treatments were paid for by the German health care insurance system.

### 2.3 Assessment of Treatment Outcome

Pre‐post symptom change was assessed with the Depression, Anxiety and Stress Scale (Lovibond and Lovibond 1995). The validity of the DASS‐21 for clinical populations (Bottesi et al. 2015) and to assess treatment outcome has been demonstrated (Ng et al. 2007; Ronk et al. 2013). It has 21 items with 4‐point Likert scales (0 = did not apply to me at all to 3 = applied to me most of the time). The three DASS subscales can be summed to a total DASS score covering general distress (Henry and Crawford 2005; Osman et al. 2012). This DASS‐21 general distress score reached excellent internal consistency (in terms of Cronbachs *α*, CR*α*) in the current sample, CR*α* = 0.937 (DASS‐21 subscales: stress CR*α* = 0.890, depression CR*α* = 0.935, anxiety CR*α* = 0.837), and was used to assess treatment outcome in the current study (for a comparable approach see von Brachel et al. 2019). As recommended for pre‐post‐treatment outcome assessment, a residual change score was calculated from the DASS‐21 general distress score in line with published recommendations (Steketee and Chambless 1992). The resulting DASS residual symptom change score covered the change in symptom load from pre to post‐treatment, with higher scores representing higher post‐treatment residual symptomatology. This pre‐post symptom measure was complemented by a two‐item global success rating (Flückiger et al. 2007; Michalak et al. 2003), used routinely at MHRTC to assess treatment outcome at the end of the treatment. It assesses patients perceived goal attainment and treatment satisfaction (6‐point scales: Have your expectations concerning this treatment been fulfilled?; range: worse than expected—completely fulfilled my expectations; Overall, how much did the treatment help you?, range: worsen the problem—helped very much); (von Brachel et al. 2019). Both items are summed to a global success score (range 2–12) (according to Michalak et al. 2003). The global success rating reached acceptable internal consistency in the current sample, CR*α* = 0.764.

### 2.4 Film Viewing Task

For the current study, two sad, two fearful, two happy, and one neutral film clips which have been shown previously to elicit the respective emotions were used (Kreibig et al. 2007; Rottenberg et al. 2002). The full description of the film clips and their validation can be found in section 2 of the Supporting Information. After a 3‐min baseline RSA measurement (rRSA) a neutral and one randomly chosen sad, one fearful, and one happy clip were presented in random order. Prior to the beginning of film viewing, participants were instructed to keep sitting quietly, to breathe regularly, to passively view the film clips, and to concentrate on the emotion they elicit. After viewing each of the four film clips, participants were asked to indicate how intensely they felt each of the six basic emotions (Ekman and Friesen 1971) while watching the clips (visual analogue scales, 0 = I did not feel the emotion at all, 100 = I extremely felt the emotion). In addition, patients rated the film clips for valence and arousal. In total, this rating procedure had a duration of approximately 30 s. Then a black screen was presented for 30 s, resulting in a total inter stimulus interval (ISI) consisting of 1 min.^2^

### 2.5 Procedure

Appointments with patients signaling willingness to participate were made prior to the beginning of the actual treatment and after the diagnostic session. Upon arrival at the laboratory, participants were seated in a comfortable recliner in a dimly lit room. Prior to the beginning of the task, all participants gave informed consent to procedures and filled in the DASS‐21. After that, electrodes were attached and the film viewing paradigm began. As part of a larger project, participants also completed an approach–avoidance task, an emotion regulation procedure, and a conditioning experiment (Adolph and Margraf 2024; Adolph et al. 2023). Upon ending of the experiment, participants indicated on 10‐point scales how demanding and arousing the experiment has been. After the experimental session, patients received treatment‐as‐usual as routinely provided at our outpatient center. After the treatment, all patients were asked to fill in the Global success rating and the DASS‐21 again.

### 2.6 Physiological Data Assessment and Data Reduction

A lead II ECG was recorded (sampling rate 1000 Hz, digitization 16 bit, Biopac MP100 amilifier system) using Ag/AgCl electrodes. A ground electrode was attached to the participant's forehead. Respiration was assessed using two respiration belts placed around the abdomen and the thorax. Additionally, electrodermal activity was recorded from the distal phalanxes of the non‐dominant hand middle and index finger. Online, data were notch filtered (50 Hz). Offline, ECG data were bandpass filtered (5‐35 Hz, 24 dB/oct), and ectopic heartbeats, as well as measurement artifacts, were corrected. After trend removal, interbeat interval data were processed through an end‐tapered Hamming Window, and FFT was applied to the data. RSA was extracted for the baseline period and the film clips as the natural logarithm of mean power within the frequency band between 0.15 and 0.40 Hz (Laborde et al. 2017). RSA parametrization was done using Kubios HRV (version, 2.1). Electrodermal and respiration data were lowpass filtered (1 Hz, 24 db/oct). Respiration rate was obtained using commercial software (Blechert et al. 2016). In addition to RSA, mean respiration rate (breaths per minute) and mean electrodermal activity (i.e., mean skin conductance level, μS) were calculated for the baseline period and while watching the four film clips. As part of a larger project, M. corrugator supercilii, M. zygomaticus major EMG were also recorded (see Adolph and Margraf 2024).

### 2.7 Data Analyses

#### 2.7.1 Diagnosis‐Based and Transdiagnostic Individual Differences in rRSA

##### 2.7.1.1 Diagnosis‐Based and Transdiagnostic Analysis of Individual Differences in rRSA

To test for differences in rRSA between diagnostic groups, an ANOVA with the between‐subject independent variable *diagnostic group* (i.e., DEP, ANX, HC) was run. rRSA (i.e., RSA during the baseline measure prior to the beginning of film viewing part) was used as the dependent variable.

To assess the transdiagnostic relationship between rRSA as well as anxiety, depression, and stress symptomatology, simple correlations were run.^3^ Afterwards, a linear regression model with the dependent variable rRSA was computed. DASS‐21 subscales showing significant associations (as substantiated by zero‐order correlations) with rRSA were entered as independent variables. To test for putative collinearity issues, we calculated the Condition Index for each of the independent variables (see Belsley et al. 1980).

Taken together, this analytical approach enabled us to assess common and distinct variance in rRSA explained by depression and anxiety diagnosis and symptomatology (e.g., Adolph and Margraf 2017).

#### 2.7.2 Individual Differences in ΔRSA

##### 2.7.2.1 Do the Film Clips Elicit Significant RSA Reactivity (ΔRSA)?

To assess if viewing the films initiated significant RSA reactivity, we ran an ANOVA with the within‐subject independent variable *condition* (i.e., resting baseline, happy, neutral, fearful, sad film clip).

##### 2.7.2.2 Diagnosis‐Based and Transdiagnostic Analyses of Individual Differences in ΔRSA

To test for individual differences in ΔRSA toward the films, changed scores were calculated between the resting baseline and the four film clips (i.e., ΔRSA = film clip– baseline).

To assess diagnosis‐based differences in ΔRSA, an ANOVA was calculated with the between‐subject independent variable diagnostic group (DEP, ANX, HC) and the within‐subject independent variable *film clip* using the four ΔRSA changed scores as dependent variables (i.e., happy, neutral, fearful, sad film clip).

To assess transdiagnostic associations of ΔRSA with depression, anxiety, and stress symptomatology, an ANCOVA was run using the same within‐subject independent variable *film clip* (i.e., the four ΔRSA changed scores for the happy, neutral, sad, fearful movies). The between‐subject independent variable diagnostic group was replaced by the continuous covariates' *anxiety* (score of the DASS‐21 anxiety subscale), *depression* (score of the DASS‐21 depression subscale) and *stress* (score of the DASS‐21 stress subscale).

##### 2.7.2.3 Additional Analyses and General Analyses Remarks

To assess the possible influence of gender and age (Voss et al. 2015) respiration (Quintana and Heathers 2014) and psychotropic medication (Hu et al. 2019) on RSA outcome measures, additional analyses including these putative confounds can be found in section 3 of the Supporting Information. As a result, confounds did not significantly impact the effects reported in this work. To assess the robustness of our statistical findings, we performed sensitivity analyses on all statistical tests using bootstrapping. We found that all results reported in our manuscript remained stable. These analyses can be found in detail in the Supporting Information.

To assess whether the effects we found for RSA measure are specific for the activity of the parasympathetic branch of the autonomic nervous system, an identical set of analyses as for RSA indices was also ran for skin conductance level data. Therefore, we compared skin conductance level during the baseline period as well as change in skin conductance level from baseline to viewing the four film clips between diagnostic groups. Also, an additional set of correlational analyses were run to assess the association of markers of sympathetic activity with the outcome of disorder‐specific CBT.

In general, for all analyses of variance, significant interactions and main effects were followed up with standard post hoc means comparisons. 90% CI calculation for *η* ^2^ effect sizes was done with the MBESS R‐package (Kelley 2007). All other statistical tests were performed with IBM‐SPSS (Version‐28) with an alpha level of 0.05.

### 2.8 Prediction of Treatment Outcome

Prediction of treatment outcome with RSA was done in two steps. In a first step, we assessed the zero‐order correlations between RSA indices (i.e., rRSA and ΔRSA toward each of the four film clips) and treatment outcome as assessed (i.e., DASS residual symptom change score and the global success rating) using Pearson correlations. To test for group differences (i.e., depression vs. anxiety) in these associations, Fisher *Z* tests were calculated.

In a second step, we conducted structural equation modeling on the ΔRSA data. With this analysis, we assessed (1) whether ΔRSA toward the different emotional film clips represents the outcome of a latent underlying process (i.e., as can be expected from theoretical considerations concering ANS functioning as outlined in the introduction section, i.e., vagal withdrawal toward meaningful emotional stimuli and situations, for prototypical changes in RSA from baseline to different emotional film clips see Fortunato et al. 2013) and (2) if a latent underlying variable ΔRSA_latent_ significantly predicts treatment outcome.

Therefore, structural equation models were estimated as follows: We first modeled a single latent variable using ΔRSA toward the four film clips as predictors. The fit of the measurement model (i.e., ΔRSA_latent_) was estimated and its fit prior to estimating structural models was ensured. CFI and TLI values greater than 0.95 and RMSEA values below 0.08 generally constitute good fit (Browne and Cudeck 1992; Little 2013). This single latent variable model fits the data well (according to the model fit indices, see results section), and was thus used to predict both treatment outcomes, i.e., the DASS residual symptom change score and the global success rating. The same structural equation model was applied to the entire group of patients and then separately to each of the two patient subgroups (DEP and ANX). All the models were conducted with IBM AMOS (Version 28).

## 3 Results

### 3.1 RSA

Of the initial participants who participated in the film viewing task, data of 2 healthy control participants, 14 depressed patients, and 11 anxiety patients were lost due to equipment malfunction. The final sample for RSA analysis thus comprised *n* = 58 healthy controls, *n* = 91 depressed patients, and *n* = 80 anxiety patients.

#### 3.1.1 Are There Diagnose‐Based Differences in rRSA?

When patient groups were defined with respect to the patient's primary diagnosis (i.e., without taking comorbidity into account) the ANOVA revealed a main effect for diagnostic group, *F*(2, 228) = 13.04, *p* < 0.001, *η* ^*2*^ = 0.103, 90% CI [0.037, 0.178]. Post hoc means comparisons showed that rRSA was higher in healthy controls as compared to patients with depression, *M*_Diff_ = 1.26, 95% CI [0.78, 1.75], *p* < 0.001, and patients with anxiety disorders, *M*_Diff_ = 0.70, 95% CI [0.20, 1.20], *p* = 0.006. Moreover, patients with anxiety disorders showed significantly higher rRSA than patients with depressive disorders, *M*_Diff_ = 0.56, 95% CI [0.12, 1.01], *p* = 0.013.

#### 3.1.2 Are There Transdiagnostic Associations With rRSA

Simple correlations indicate that higher depression, *r* = −0.246, *p* < 0.001, and anxiety symptom load, *r* = −0.207, *p* = 0.002, but not stress symptomatology, *r* = −0.068, *p* = 0.309, were associated with less rRSA. The regression analysis simultaneously including depression and anxiety symptoms as predictors revealed a significant regression equation, *F*(1,224) = 7.87, *p* < 0.001, *R* ^*2*^ = 0.066. Less rRSA was significantly associated with higher symptom load on the depression, *β* = −0.191, *p* = 0.019, (Condition Index = 3.9), but not on the anxiety subscale, *β* = −0.093, n.s., (Condition Index = 3.1).

#### 3.1.3 Do the Film Clips Elicit Significant RSA Reactivity as Compared to the Resting Baseline (ΔRSA)?

**FIGURE 1:**
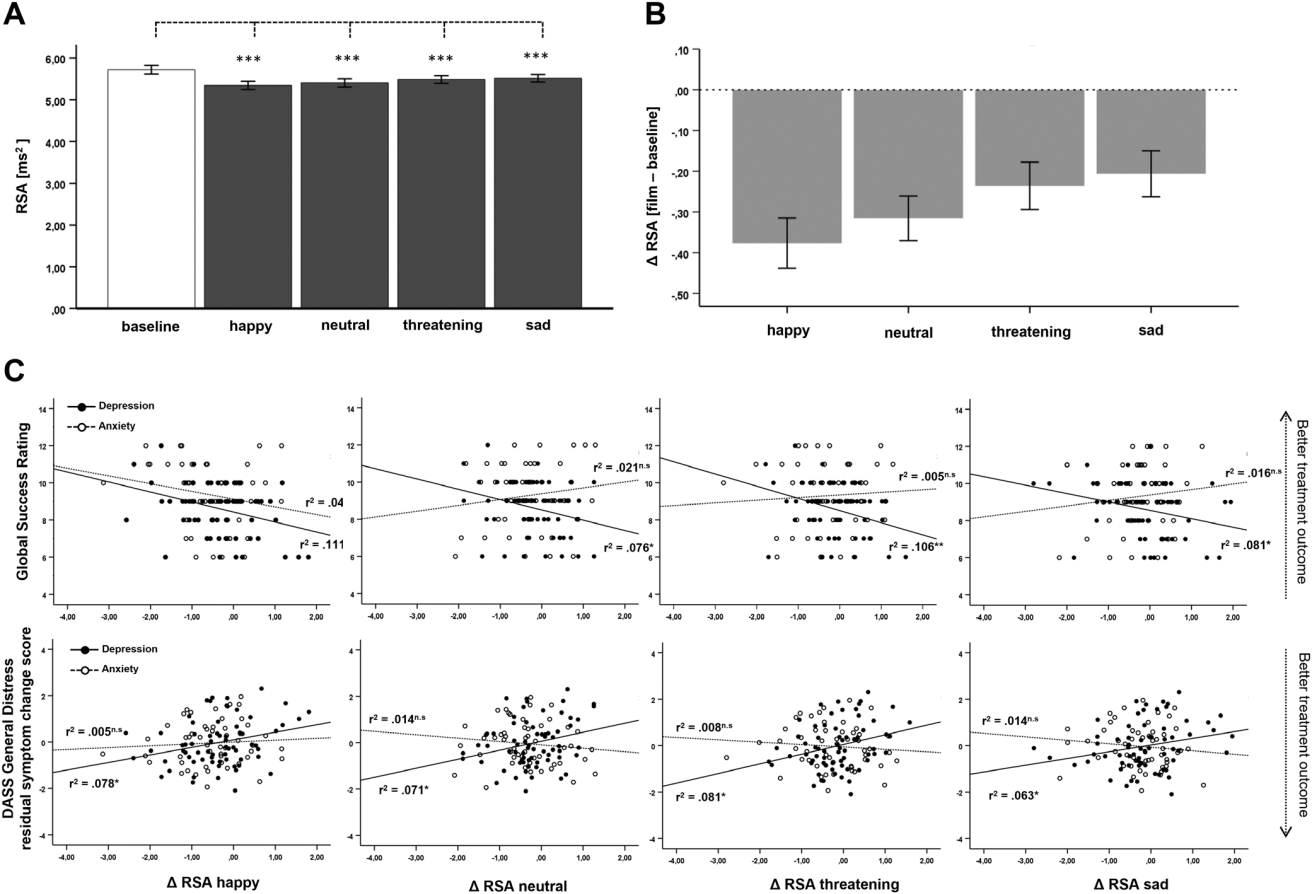
(A) RSA level during baseline and while viewing the four film clips. (B) RSA change from baseline (i.e., ΔRSA, film clip—baseline) for the four film clips. (C) Scatterplots for the correlations between ΔRSA and treatment outcome for the global success rating (upper panel) and the DASS general distress residual symptoms change score (lower panel). Bold lines represent regression lines for the group of depressed patients, and dotted lines for the anxiety patients.

Figure 1 (A) left panel gives an overview of RSA levels at rest and while viewing the four film clips. As expected, the ANOVA revealed a significant main effect for condition, *F*(4,876) = 16.33, *p* < 0.001, *η* ^2^ = 0.069, 90% CI [0.042, 0.094]. Standard means comparisons revealed that RSA was significantly higher during the resting baseline as compared to all of the four film clips (Resting Baseline vs. Happy: *M*_Diff_ = 0.38, 95% CI [0.26, 0.49], *p* < 0.001; Resting Baseline vs. Neutral: *M*_Diff_ = 0.31, 95% CI [0.21, 0.42], *p* < 0.001; Resting Baseline vs. Fear: *M*_Diff_ = 0.23, 95% CI [0.12, 0.34], *p* < 0.001; Resting Baseline vs. Sad: *M*_Diff_ = 0.20, 95% CI [0.09, 0.31], *p* < 0.001).

#### 3.1.4 Are There Diagnose‐Based Differences in ΔRSA?

ΔRSA differed significantly between the four film clips (see Figure 1B) as qualified by a significant main effect for film clip, *F*(3, 651) = 5.78, *p* < 0.001; *η* ^2^ = 0.026, 90% CI [0.005, 0.046]. Post hoc means comparisons showed that ΔRSA was largest for the happy film clip, differing significantly from the threatening, *M*_Diff_ = −0.14, 95% CI [−0.22, −0.06], *p* < 0.001 and sad film clip, *M*_Diff_ = −0.17, 95% CI [−0.26, −0.08], *p* < 0.001. Furthermore, ΔRSA differed significantly between the neutral and the sad film clip, *M*_Diff_ = −0.11, 95% CI [−0.20, −0.01], *p* = 0.025. There were no more significant differences in ΔRSA between the film clips.

There were no differences in ΔRSA between the diagnostic groups as indicated by a non‐significant between‐subject independent variable diagnostic group, *F*(1, 217) = 1.29, *p* = 0.276, and a non‐significant interaction film clip × diagnostic group, *F*(6, 651) = 0.55, *p* = 0.770.

#### 3.1.5 Are There Transdiagnostic Associations With ΔRSA

The ANCOVA revealed that overall, less ΔRSA was significantly associated with higher depression symptomatology, *F*(1, 214) = 8.32, *p* = 0.004; *η* ^2^ = 0.037, 90% CI [0.007, 0.087] but not significantly associated with anxiety, *F*(1, 214) = 0.02, n.s., or stress symptomatology, *F*(1, 214) = 3.58, n.s. There were no significant interactions between the within‐subject independent variable film clip and the depression, anxiety, or stress scales of the DASS.

#### 3.1.6 Prediction of Treatment Outcome With Markers of RSA

Data from *n* = 77 patients with depressive and *n* = 60 patients with anxiety disorders were available for analyses. The remaining patients either did not attend the post‐treatment questionnaire assessment (*n* = 23) or prematurely terminated their treatments (*n* = 27). Data of 9 patients were lost due to technical reasons. Importantly, patients who dropped out did not differ from those who continued the study in their gender, age, nor in their depression, anxiety, or stress symptoms scores (all *p* > 0.10).

Table 2 gives an overview of significant simple associations between treatment outcome and rRSA as well as ΔRSA toward the different film clips. In sum, rRSA was not significantly associated with the two treatment outcome measures, neither for depressed nor for patients with anxiety disorders. On the contrary, enhanced ΔRSA toward all of the four film clips (i.e., more RSA withdrawal) was associated with better treatment outcome on both outcome measures—that is, DASS residual symptom change score (see Figure 1C lower panel) and the global success rating (see Figure 1C upper panel)—in the group of depressed patients, but not in the group of anxiety patients. Moreover, Fisher *Z* tests confirm that, with the exception of ΔRSA toward the happy film clip, all correlation coefficients were significantly larger in the depression group, as compared to the anxiety group (see Table 2).

**TABLE 2:** Correlations of RSA indices with the post‐treatment global success rating (upper part) and DASS general distress residual symptom change score (lower part) for depressed patients and anxiety patients.

	Depression	Anxiety	Group comparison
	*r*	*r*	*Z*
*Post‐treatment global success rating*			
rRSA	0.093^n.s.^	0.193^n.s.^	−0.54^n.s.^
ΔRSA_hap_	−0.322^*p* < 0.01^	−0.226^n.s.^	−0.54^n.s.^
ΔRSA_neut_	−0.258^*p* < 0.05^	0.107^n.s.^	−1.95^*p* < 0.05^
ΔRSA_threat_	−0.310^*p* < 0.05^	0.036^n.s.^	−1.88^*p* < 0.05^
ΔRSA_sad_	−0.269^*p* < 0.05^	0.083^n.s.^	−1.89^*p* < 0.05^
*DASS general distress residual symtom change score*			
rRSA	−0.056^n.s.^	−0.135^n.s.^	0.42^n.s.^
ΔRSA_hap_	0.268^*p* < 0.05^	0.095^n.s.^	0.94^n.s.^
ΔRSA_neut_	0.250^*p* < 0.05^	−0.086^n.s.^	1.80^*p* < 0.05^
ΔRSA_threat_	0.269^*p* < 0.05^	−0. 062^n.s.^	1.78^*p* < 0.05^
ΔRSA_sad_	0.236^*p*=0.05^	−0.080^n.s.^	1.69^*p* < 0.05^

Abbreviations: hap, happy film clip; neut, neutral film clip; n.s., non‐significant; sad, sad film clip; threat, threatening film clip.

**FIGURE 2:**
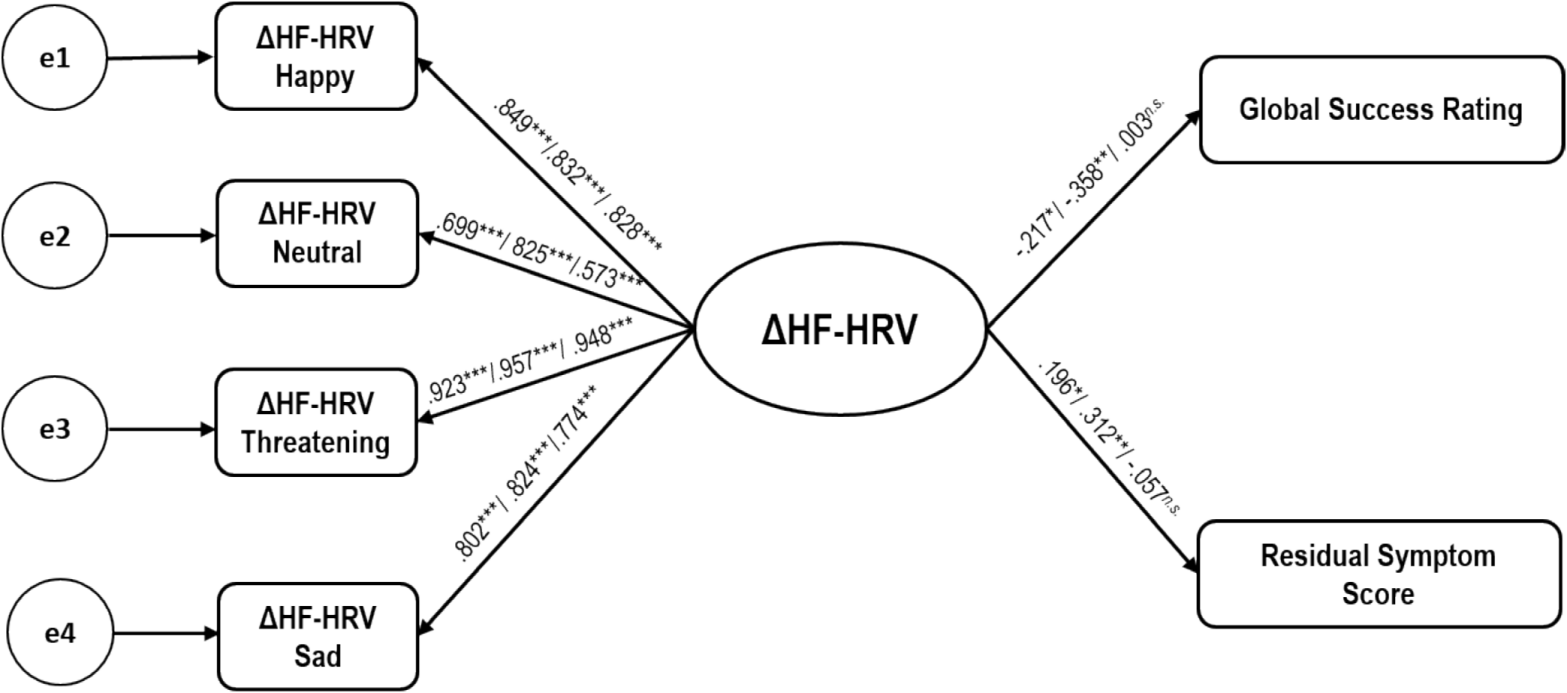
Structural equation model for the prediction of treatment outcome with the DASS general distress residual symptoms change score and the global success rating. ***p < 0.001, **p < 0.01, * p < 0.05; Standardized regression coefficients for the entire group of patients are given on the arrows as follows: all patients/depressed patients/anxiety patients. n.s.= non significant.

To test whether ΔRSA toward the four film clips represents independent processes or is all the outcome of a single underlying process, we performed structural equation modeling. We first modeled a single latent variable using ΔRSA toward the four film clips as predictors. As a result, we found that the resulting measurement model for the ΔRSA_latent_ variable fits the data very well, *χ* ^*2*^(2) = 0.779, *p* = 0.677, CFI = 1.000, RMSEA = 0.000, 90% CI [0.000–0.107]. In a second step, the latent model (see Figure 2) was conducted separately for the entire group of patients and for the depression and anxiety groups alone using ΔRSA_latent_ as predictor and the two treatment outcome measures as dependent variables. For the entire group of patients (i.e., depression and anxiety), more pronounced RSA withdrawal toward the film clips in terms of smaller ΔRSA values predicted better treatment outcomes in terms of larger global success ratings (*β* = −0.217, *p* = 0.020) and less post‐treatment residual general distress (i.e., a smaller DASS residual symptom change score, *β* = 0.196, *p* = 0.037).

This pattern was evident also for the depressed patients alone (global success ratings, *β* = −0.358, *p* = 0.002, DASS residual symptom change score, *β* = 0.312, *p* = 0.009). However, for anxiety patients alone, latent ΔRSA neither significantly predicted the global success rating (*β* = 0.003, *p* = 0.958) nor the DASS residual symptom change score (*β* = −0.057, *p* = 0.692).

### 3.2 Skin Conductance Level

#### 3.2.1 Individual Differences in Skin Conductance Level During Baseline

We did not find any differences between the diagnostic groups in Skin Conductance Level during the resting baseline condition, *F*(2, 222) = 1.40, *p* = 0.250.

#### 3.2.2 Skin Conductance Level During Film Clip Viewing

As evidenced by a significant main effect for condition, *F*(4,872) = 44.05, *p* < 0.001, *η* ^2^ = 0.168, 90% CI [0.129, 0.202], participants exhibited significantly higher skin conductance level while viewing the film clips as compared to the baseline condition. Single post hoc means comparisons indicate that this was true for the happy, *M*_Diff_ = −0.64, 95% CI [−0.76, −0.52], *p* < 0.001, neutral, *M*_Diff_ = −0.59, 95% CI [−0.71, −0.48], *p* < 0.001, threatening, *M*_Diff_ = −0.61, 95% CI [−0.73, −0.49], *p* < 0.001, and sad film clip, *M*_Diff_ = −0.61, 95% CI [−0.77, −0.45], *p* < 0.001. There was no significant main effect for diagnostic group, *F*(2,218) = 0.99, *p* = 0.372; nor a significant interaction for diagnostic group × condition, *F*(8,872) = 0.59, *p* = 0.688.

#### 3.2.3 Prediction of Treatment Outcome With Skin Conductance Level

We did not find any significant associations between skin conductance level during baseline or skin conductance change from baseline to viewing the film clips and any treatment outcome measure (all *p* > 0.10).

## 4 Discussion

Here, we assessed (1) the significance of resting Respiratory Sinus Arrhythmia (rRSA) and Respiratory Sinus Arrhythmia reactivity (ΔRSA), two markers of cardiac vagal control for the mood and anxiety disorders spectrum as well as (2) the predictive validity these markers have for the outcome of disorder‐specific CBT. In line with our hypotheses, the group‐based approach demonstrated that patients regardless of diagnosis exhibited significantly lower rRSA than healthy controls. However, transdiagnostically, we found a more robust association of RSA indices with depression symptomatology. In specific, depression, but not anxiety symptomatology was transdiagnostically associated with less favorable rRSA and ΔRSA. Furthermore, confirming a more robust association between depression symptomatology and RSA, the current study shows for the first time that more favorable ΔRSA is predictive of more favorable treatment outcome in depression, but not in anxiety. Importantly, we did not find any effect for skin conductance level, a physiological marker specifically reflecting the activity of the sympathetic branch of the autonomous nervous system (ANS). This indicates specificity of the current findings for the activity of the parasympathetic branch of the ANS. In addition, these current results were not affected by interindividual differences in respiration, psychotropic medication, age, or gender.

### 4.1 Associations Between RSA Indices and Psychopathology (Cross‐Sectional Approach)

As expected, the group‐based analysis showed that patients with depressive and anxiety disorders displayed significantly lower rRSA than healthy controls. This finding is in line with meta‐analyses showing that internalizing disorders (Chalmers et al. 2014; Koch et al. 2019) are associated with reduced rRSA. As stated in the neurovisceral integration model (Thayer and Lane 2000, 2009) high levels of rRSA reflect the activity of a top‐down regulation system (Beauchaine 2015; Thayer et al. 2012) involved in the organism's flexible physiological reactivity to emotional or stress‐related environmental demands. rRSA has been previously associated with behavioral flexibility, cognitive executive functioning, and self‐regulation (Allen et al. 2000; Hansen et al. 2009; Thayer and Lane 2000), processes frequently impaired in mood and anxiety disorders (Aldao et al. 2010). Moreover, following the neurovisceral integration model, RSA is associated with activation of the medial prefrontal cortex (Thayer et al. 2012), a heterogeneous neural structure critically involved in a wide range of emotional processes (Schneider and Koenigs 2017). Our data are thus in line with previous research attributing impairments in prefrontal cortex functioning to both disorders (e.g., Hiser and Koenigs 2018).

Despite the overall significance of RSA for disorders from the depressive and anxiety disorders spectrum, its differential relationship with depression and anxiety symptomatology seems to be more complex. Indeed, although the group‐based analyses indicate that both patients with anxiety and depression exhibit reduced rRSA, the transdiagnostic analysis (across the entire depressive and anxiety disorders spectrum and healthy controls) simultaneously including questionnaire‐based depression and anxiety symptomatology as predictors showed that higher symptoms of depression, but not anxiety, predicted lower rRSA. That is, depression symptomatology explained most of the variance anxiety symptomatology shared with rRSA. This clearly suggests a more robust transdiagnostic association between depressive symptomatology and rRSA in a direct comparison with anxiety symptoms. This specific association of rRSA with depression symptomatology is paralleled by the ΔRSA results. That is, also less ΔRSA was transdiagnostically (i.e., across the entire sample) associated with higher depression—but not anxiety symptomatology. This association was not restricted to the sadness‐inducing film clip, but rather broadly distributed over the full range of emotional clips. This indicates that broad blunted ΔRSA is specifically associated with more severe depression symptomatology. These data thus are in line and extend current meta‐analyses associating depression (Hamilton and Alloy 2016; Schiweck et al. 2019) but not anxiety (Campbell and Wisco 2021) with less favorable ΔRSA. Paralleling this, while depression and anxiety share a liability to experience negative affect, both disorders are distinguishable on the basis of their emotional reactivity (Mineka et al. 1998; Shankman and Klein 2003). That is, an impressive number of studies show that most (but not all) anxiety disorders are related to processing deficits toward specific, individually fear relevant stimuli (Amin et al. 1998; Dillon et al. 2014; Hamm et al. 1997; Hermann et al. 2009; In‐Albon et al. 2008; Joiner et al. 1999; McTeague and Lang 2012; Paul et al. 2016; Shankman and Klein 2003; Wannemueller et al. 2017; Woud et al. 2014). In contrast, indicating sustained context insensitivity in depression (e.g., Bylsma 2021), depression is related to broad blunted affective reactivity toward a diverse range of emotional challenges (Bylsma et al. 2008; Gehricke and Shapiro 2000; Rottenberg et al. 2002; Rottenberg, Salomon, et al. 2005; Sloan et al. 1997; Sloan et al. 2001), including reactivity toward neutral stimulus materials (Granros et al. 2022; Nelson et al. 2015). This striking link between broad blunted ΔRSA in the current study and blunted affective reactivity as shown in the literature is further supported by findings indicating that ΔRSA predicts subjective responses toward film‐based mood inductions (Beevers et al. 2011). Moreover, in general, RSA is dependent on the momentary amount of cognitive involvement, whereby more cognitive involvement is associated with higher vagal reactivity (Durantin et al. 2014; Hidalgo‐Muñoz et al. 2018; Muth et al. 2012). Taken together, the current data further support and extend previous findings on emotion context insensitivity in depression. They clearly highlight a specific association of ECI with depression symptomatology rather than anxiety symptoms and at the same time show the transdiagnostic relevance emotion context insensitivity has across a naturalistic comorbid sample of patients from the internalizing symptoms spectrum. Thus, the current data might suggest the usefulness of ΔRSA as a transdiagnostic biomarker for depression‐symptom‐based ECI across the mood and anxiety disorders spectrum.

### 4.2 Associations Between RSA Indices and Treatment Outcome (Longitudinal Approach)

Further substantiating the specific associations we found between depression symptomatology and RSA, our data show specific predictive validity of ΔRSA for treatment outcome in patients with depression. In fact, more favorable ΔRSA (more RSA withdrawal) specifically predicts better treatment outcome in depression (i.e., less post‐treatment symtoms as measured with the DASS, as well more subjective treatment satisfaction, as assessed with the global success rating). No such association was found for anxiety disorders. Only very few studies so far investigated the predictive validity of ΔRSA. Rottenberg and colleagues (Rottenberg, Gross, et al. 2005) showed that more pronounced vagal withdrawal to a sad film predicts recovery from depression and less symptom severity at 6‐month follow‐up, while others (Tolin et al. 2021) did not find any association between ΔRSA and the outcome of a CBT intervention in anxiety disorders. Our data complement this work and show for the first time specific predictive validity of ΔRSA for the outcome of multi‐session CBT treatments in depressive disorders. It has to be determined if the current findings reflect the physiological readout of a specific mechanism in depression treatments. The fact that there was no association between ΔRSA and symptom reduction from pre to post‐treatment in the anxious group might suggest that the predictive relationship between ΔRSA and treatment outcome in depression is not solely based on spontaneous symptom recovery. Moreover, as compared to anxiety, emotional or regulative processes are among the core mechanisms of change within treatments of depressive disorders, while anxiety treatment frequently targets exposure techniques, with extinction learning as a key mechanism of change (Adolph et al. 2023; Kazantzis et al. 2018; Powers et al. 2017). In line with this, it has been shown that CBT enhances global cognitive control network activity in depression (Yang et al. 2018) as well as emotion regulation abilities (Forkmann et al. 2014), processes which have been previously associated with RSA. Moreover, our data support previous findings that broad improvement of affect regulation capacity, including adaptation processes toward emotion stimulation and their neuronal underpinnings, might be specific mechanisms of change within depression‐specific CBT (Forkmann et al. 2014; Ritchey et al. 2011). Especially in line with this, predictive validity of ΔRSA was again not restricted to vagal withdrawal toward the “disorder‐specific” sadness‐inducing stimulus only (Rottenberg, Salomon, et al. 2005; Yaroslavsky et al. 2013). In contrast, our data indicate that more favorable vagal withdrawal toward all movies predicted better treatment outcome. Again, these data are clearly in line with the predictions of the emotion context insensitivity theory (Bylsma 2021; Rottenberg, Gross, et al. 2005) and indicate that less context insensitivity in vagally mediated emotional responses predicts better CBT outcome. Thus, supporting previous reports of improvements in vagally mediated heart rate variability after a CBT intervention in depression (Carney et al. 2000) the current data further point to ΔRSA as a specific marker for treatment change in depression. However, clearly further research is needed to substantiate this specific predictive validity of ΔRSA as a physiological indicator of emotion adaptation processes as a depression‐specific mechanism in mood disorders.

### 4.3 Transdignostic Relevance of the Current Findings

The remarkable dissociations between our group‐based and our transdiagnostic analyses in both rRSA and ΔRSA speak in favor of recent attempts to develop psychometrically robust dimensional classification systems like the hierarchical taxonomies of mental disorders (HiTop, Kotov et al. 2017) or the Research Domain Criteria Framework (RDoC, Cuthbert 2014). A transdiagnostic approach might be more sensitive to detect subtle interindividual differences in heterogeneous samples like the current one yielding high comorbidity rates. However, overall the effect sizes were comparably small and thus our data should be interpreted with some caution. They nonetheless can serve as starting points for future research into the common and distinct associations between RSA and depression and anxiety. Our data further support current theoretical models (Beauchaine 2015) highlighting the usefulness of RSA as a transdiagnostic biomarker cutting across traditional diagnostic categories. They thus complement previous research showing a transdiagnostic association of RSA with suicidal ideation (Adolph et al. 2018). Furthermore, in showing a common association between RSA across depression and anxiety disorders, our data is in line with the idea of a general factor underlying the mood and anxiety disorders spectrum (Beauchaine 2015; Kotov et al. 2017), and are broadly compatible with the ideas of the Research Domain Criteria (Cuthbert 2014), an initiative to identify transdiagnostic biomarkers for specific cognitive or emotional processes which operationalize mental disorders to fall along a continuum ranging from normal to disordered functioning (Cuthbert 2014). For example, the RDoC Arousal construct describes the organism's sensitivity toward external and internal stimuli. Thereby, the organism's ability to generate a context‐appropriate amount of arousal facilitates interaction with the environment in a context‐specific manner (see Kozak and Cuthbert 2016). We found lower rRSA (indicating lower cardic vagal control) and blunted ΔRSA (indicating less cardiac vagal reactivity) to be transdiagnostically associated with various symptoms from the internalizing disorders spectrum. These data add to previous research on the RDoC Arousal construct associating internalizing spectrum symptomatology to deviations in markers of sympathetic arousal, including pupil dilation, cortisol response, or skin conductance (McCord et al. 2017; Kircanski et al. 2017; Panayiotou et al. 2017), and suggest markers of RSA as a useful complement to assess the transdiagnostic relevance of the RDoC Arousal construct.

### 4.4 Caveats

Our study comes with limitations. First, we recruited a naturalistic, ecologically valid sample of patients receiving treatment‐as‐usual in our outpatient center. Thus, closely monitoring the therapeutic process, as usually conducted in randomized controlled trials, was not possible. Enhanced ecological validity of the current samples and the treatment process most likely caused limitations in treatment integrity. However, as mentioned above, treatments in our center regularly follow published Cognitive Behavior Therapy manuals, and treatment sessions are monitored regularly by trained supervisors (von Brachel et al. 2019) assuring general adherence to treatment manuals. Moreover, although disorder‐specific manuals are used, all manuals cover Cognitive Behavior Therapy, and all anxiety manuals are comparable in being exposure‐based. Second, a considerable number of patients did not attend the post‐treatment questionnaire assessment or terminated the treatment prematurely (see results section for details). This resulted in a considerable reduction in the power of our analyses concerning the prediction of treatment outcome. However, the current response rate is comparable to previous studies in outpatient settings (von Brachel et al. 2019), and patients who finished the study and those who dropped out did not differ from each other in terms of rRSA, ΔRSA, gender, age, or their depression, anxiety, or stress level. Moreover, because we recruited a large patient sample, the final *N* for the prediction of treatment outcome was still considerably high (*n* = 135), nonetheless yielding considerable statistical power. Thus, it is unlikely that the current effects were severely affected by the current dropouts.

Third, since the current study had been started before the final release of the DSM‐5 in Germany, the now outdated DSM‐IV classification system has been used in the present study. As a result, the current anxiety sample includes patients with obsessive compulsive disorder as well as with posttraumatic stress disorder, which within the DSM‐5 are no longer considered anxiety disorders. However, we carried out a set of analyses excluding patients with an OCD and a PTSD diagnosis. In fact, the results yielded results similar to the original analyses carried out including the OCD and PTSD patients. Thus, the inclusion of these patients did not significantly bias the current findings, and our control analyses assure that the current results are valid also for diagnoses obtained with the current DSM‐5 disorder categories.

Clearly, future studies are needed to clarify if the more pronounced association between RSA and depression is a function of the depression symptomatology per se, or of higher symptom load in the depressed sample. Indeed, a more severe burden of disease has been attributed to depression as compared to a multitude of somatic widespread diseases (Murray and Lopez 2013) or other mental disorders including anxiety (Whiteford et al. 2013). As depression and anxiety are often accompanied by significant dysregulation of the stress system (Gold 2015; Heim and Nemeroff 2001) they have an enhanced risk of developing cardiovascular diseases (CVD, Steptoe and Kivimäki 2012). Stress and CVD are themselves associated with lowered heart rate variability (Hillebrand et al. 2013), complicating the interpretation of the current findings. The high comorbidity between depression and anxiety disorders, in addition to the fact that some anxiety disorders are more likely to precede the onset of depression while others onset secondary to depression (Fava et al. 2000), underscores the need for further studies to disentangle the putatively intertwined mechanisms underlying the association between psychopathological states and RSA. Finally, we did not assess income, education, and socioeconomic status. This putatively hampers the generalizability of the current data.

## 5 Conclusion

Taken together, the current data support theoretical considerations on the significance of indices of RSA as transdiagnostic biomarkers relevant for the mood and anxiety disorders spectrum. At the same time, our data provide convincing first evidence that this transdiagnostic association is more pronounced for depression‐ rather than anxiety‐ symptomatology. This highlights the importance of recognizing symptom comorbidity within experimental approaches toward mechanisms underlying the mood and anxiety disorders spectrum. Moreover, the current data show that mechanistic transdiagnostic approaches are more sensitive for detecting interindividual differences in naturalistic clinical samples. Thus, the current findings clearly support current attempts to develop psychometrically robust, transdiagnostic, and dimensional clinical measures, like the hierarchical taxonomies of mental disorders (HiTop, Kotov et al. 2017) or the Research Domain Criteria Framework (RDoC, Cuthbert 2014) allowing for an ecologically valid assessment of psychopathology and overcoming known issues with current nosologies (Michelini et al. 2021). Furthermore, our data suggest a more robust transdiagnostic association of (at least) ΔRSA with depression and thus suggest that rRSA and ΔRSA constitute potential transdiagnostic biomarkers enabling the assessment of common *and* distinct emotion‐related mechanisms associated with the depression and anxiety spectrum.

## Author Contributions

**Dirk Adolph:** conceptualization, data curation, formal analysis, investigation, methodology, writing – original draft. **Xiao Chi Zhang:** formal analysis. **Tobias Teismann:** resources, writing – review and editing. **Andre Wannemüller:** writing – review and editing. **Jürgen Margraf:** conceptualization, methodology, supervision, writing – review and editing.

## Conflicts of Interest

The authors declare no conflicts of interest.

## Supporting information


Data S1.

## Data Availability

The data will be made available upon reasonable request.

## Endnotes

^1^
The current sample of patient is representative for the entire patient population attending for treatment at our outpatient center during the period of data assessment for the current study in terms of distribution of diagnoses, *χ* ^*2*^_(2)_ = 2.67, *p* = 0.263, gender, *χ* ^*2*^_(2)_ = 0.23, *p* = 0.629, and age, *t*(1827) = 0.80, *p* = 0.425.

^2^
An interstimulus interval of 1 min has been shown to be sufficient for the recovery of vagal activity after exposure to a psychological stressor (e.g., Mezzacappa et al. 2001).

^3^
After reviewing extensive literature, Clark and Watson (1991) found that anxiety and depression although consisting of specific, unique features, share a common component of negative affect/distress. They proposed a tripartite model consisting of three components: negative affect (common to both anxiety and depression), physiological hyperarousal (unique to anxiety), and low positive affect (unique to depression). Although the DASS was developed independent of this model, its three distinct factors are suppost to align with this tripartite structure (Brown et al. 1997). Specifically, DASS‐Depression reflects low positive affect, DASS‐Anxiety captures physiological hyperarousal, and DASS‐Stress corresponds to general negative affect/stress (Brown et al. 1997). Thus, to assess the entire range of symptomatology from the mood and anxiety spectrum, depression, anxiety and stress were included in the analysis.
