# Supplementary material for: Respiratory Sinus Arrhythmia—Common and Distinct Mechanisms of Emotional Adjustment in the Depressive and Anxiety Disorders Spectrum?
Source: Psychophysiology. 2025 May 31;62(6):e70079. doi: 10.1111/psyp.70079 (PMC12125617; doi:10.1111/psyp.70079)
Supplement: Supplementary file 1 — Data S1. [file PSYP-62-e70079-s001.docx]

1. **Manuals used for treatment at Mental Health Research and Treatment Center**

***Anxiety Disorders manuals (all manuals cover exposure-based Cognitive Behvavior Therapy)***

*Social Anxiety Disorder:* Stangier,U., Clark, D.M., Ginzburg, D.M. & Ehlers, A. (2016). Soziale Angststörung. Göttingen: Hogrefe.
*Panic and agoraphobia:* Schneider, S. & Margraf, J. (2017). Agoraphobie und Panikstörung. Göttingen: Hogrefe.
*Specific Phobia:* Hamm, A. (2006). Spezifische Phobie. Göttingen: Hogrefe.
*Somatisation and Hypochondriasis:* Rief, W. & Hiller, W. (1998). Somatisierungsstörung und Hypochondrie. Göttingen: Hogrefe.
*Generalised Anxiety Disorder:* Becker, E. & Margraf, J. (2016). Generalisierte Angststörung. Göttingen: Hogrefe.
*Post Traumatic Stress Disorder:* Foa, E.B., Hembree, E.A., & Rothbaum, B.O. (2017). Prolonged Exposure Therapy for PTSD. Lichtenau: Probst.
*Obsessive Compulsive Disorder:* Lakatos, A. & Reinecker, H. (2016). Kognitive Verhaltenstherapie bei Zwang. Göttingen: Hogrefe.

***Depression manual (covering Cognitive Behavior Therapy for Depression)****:*

Hautzinger, M. (2021) Kognitive Verhaltenstherapie der Depression. Weinheim: Beltz-Verlag*.*

1. **Film Clip validation**

For the current study, two sad, two threatening, two happy, and one neutral film clips were taken from famous motion pictures (sadness: The Champ, 2:38 min, MGM, United Artists film studios, 1979; My sister’s keeper, 3:36 min, Warner Bros, Paramount, 2009, threat: Halloween, 3:28 min, 1978, Compass International Pictures, The silence of the lambs, 3:22 min, 1991, Orion Pictures, happiness: 500 days of summer, 2009, 1:46 min, Searchlight Pictures, An officer and gentleman, 1:51 min, Paramount 1982; neutral: All the president’s men, 1:05 min, 1976, Wildwood Enterprises). All clips have been shown previously to elicit the respective emotions (e.g. Kreibig et al., 2007; Rottenberg et al., 2002)

To assess whether the two sadness, happiness and threat-eliciting film clips differ in their potency of eliciting the respective target emotion, t-tests were run (i.e. sadness: The Champ vs. My Sisters Keeper, threat: Helloween vs. The Silence of the Lambs, happiness: 500 Days of Summer vs. An Officer and Gentleman). These analyses showed that the two happy, sad, and fearful clips did not differ in their potency to elicit sadness (The champ: *M*=73.74, *SD*=26.32; My sisters keeper: *M*=77.03, *SD*=23.41, *t*(244) = 1.00, *p* = .319), fear (Halloween: *M*=56.32, *SD*=32.49; The silence of the lambs: *M*=50.74, *SD*=29.72, t(248) = 1.35, *p* = .179) or happiness (500 days of summer: *M*=69.18, *SD*=28.86; An officer and gentleman: *M*=67.56, *SD*=30.19, *t*(241) = 0.43, *p* = .670). To assess whether the emotional film clips elicited more intense emotions than the neutral film, t-tests were run comparing the mean sadness, fear, and happiness ratings towards the emotional clips and the neutral film clip. Importantly, the emotional clips elicited more intense target-emotions than the neutral film clip (i.e. *happiness*: happy vs. neutral: *M_diff_* =50.20, 95%CI (45.70, 54.79), *t*(249)=21.96 *p*<.001; *sadness*: sad vs. neutral *M_diff_* =66.82, 95%CI (63.14, 70.49), *t*(249)=35.84, *p*<.001; *fear*: fearful vs. neutral: *M_diff_* =43.75, 95%CI (39.61, 47.90), *t*(249)=20.80, *p*<.001).

1. **Analyses controlling for gender, age, respiration and psychotropic medication on interindividual differences in RSA**

In a first step, we assessed whether any of the putative influencing factors (gender, age, respiration and psychotropic medication) were associated with the two outcome measures (rRSA, ΔRSA) using Pearson correlations. rRSA correlated significantly with medication, *r*=-.295, *p*<.01, gender, *r*=.179, *p*<.01, and age, *r*=-.460, *p*<.01. ΔRSA correlated significantly with change in respiration rate from baseline to watching the emotional videos (ΔRR), *r*=-.359, *p*<.01 and gender, *r*=-.143, *p*<.05. To control for these potential confounding factors, a set of control analyses were run.

**3.1 rRSA**

To control for the influence of medication, sex and age an ANCOVA including age as continuous covariates, as well as gender (male, female), psychotropic medication (yes, no) and diagnostic group (ANX, DEP, HC) as between subject independent variables was calculated. To control for these putative influencing factors within our transdiagnostic analyses, stepwise linear regression models were calculated with rRSA as dependent variable.

**Table S1:** ANCOVA controlling for the influence of Age, Gender and Medication on group-based individual differences in rRSA

| **Effect** | ***DF*** | ***F*** | ***p*** |
| --- | --- | --- | --- |
| Age | 1, 229 | 55.37 | <.001 |
| **Diagnostic Group** | **2, 229** | **4.18** | **.017** |
| Gender | 1, 229 | 3.77 | .053 |
| Medication | 1, 229 | 7.83 | .006 |
| Diagnostic Group x Gender | 2, 229 | 2.75 | .066 |
| Diagnostic Group x Medication | 1, 229 | 0.77 | .384 |
| Gender x Medication | 1, 229 | 0.56 | .454 |
| Diagnostic Group x Gender x Medication | 1, 229 | 1.17 | .280 |

Note: Bold lines indicate significant effects within the analyses reported in the main manuscript

**Table S2**. Regression controlling for the influence of age, gender and medication on transdiagnostic individual differences based on depression symptomatology in rRSA

|  | | | | ***β*** | ***t*** | ***p*** |
| --- | --- | --- | --- | --- | --- | --- |
| **Step 1** | | ***F (1, 224) = 58.78, p<.001, R^2^=.209*** | | | | |
|  | | Age | | -.457 | -7.67 | <.001 |
| **Step 2** | | ***F (2, 224) = 40.68, p<.001, ΔR^2^=.057*** | | | | |
|  | | Age | | -.428 | -7.41 | <.001 |
|  | | Medication | | -.246 | -4.25 | <.001 |
| **Step 3** | ***F (3, 224) = 31.00, p<.001, ΔR^2^=.025*** | | | | | |
|  | Age | | -.424 | | -7.46 | <.001 |
|  | Medication | | -.204 | | -3.48 | <.001 |
|  | **DASS_Depression_** | | **-.173** | | **-2.96** | **.003** |
| **Step 4** | ***F (4, 224) = 25.08, p<.001, ΔR^2^=.014*** | | | | | |
|  | Age | | -.423 | | -7.51 | <.001 |
|  | Medication | | -.195 | | -3.35 | <.001 |
|  | **DASS_Depression_** | | **-.160** | | **-2.77** | **.006** |
|  | Gender | | .131 | | -2.33 | .021 |

Note: Bold lines indicate significant effects within the analyses reported in the main manuscript. Rejected variable in final model (Step4): DASS Anxiety β = -.105, p=.133

**3.2 ΔRSA**

Gender and respiration rate were significantly associated with ΔRSA. For the analysis of individual differences in ΔRSA based on diagnostic groups, ANCOVA including gender (male, female), and diagnostic group (ANX, DEP, HC) as between subject independent variables, as well as respiration rate as continuous covariate was calculated.

To control for these putative influencing factors within our transdiagnostic analysis, an ANCOVA was conducted with ΔRSA as dependent variable. The between subject independent variables diagnostic group (ANX, DEP, HC) and gender (male, female), and the within subject independent variable film clip (happy, neutral, sad, fear) were included. Finally, ΔRR was entered as a continuous covariate to the model.

**Table S3:** ANCOVA controlling for the influence of Gender and Respiration on individual differences in ΔRSA

| ***Group Based Analysis*** | | | |
| --- | --- | --- | --- |
| **Effect** | ***DF*** | ***F*** | ***p*** |
| Gender | 1, 212 | 2.82 | .094 |
| Diagnostic Group | 2,212 | 1.86 | .158 |
| Gender x Diagnostic Group | 2,212 | 1.35 | .261 |
| **Film Clip** | **3, 636** | **4.59** | **.003** |
| Film Clip X ΔRR | 3, 636 | 2.61 | .051 |
| Film Clip X Diagnostic Group | 6, 636 | 0.76 | .603 |
| Film Clip X Gender X Diagnostic Group | 6, 636 | 1.55 | .160 |
| ***Transdiagnostic Analysis*** | | | |
| Gender | 1, 212 | 4.44 | .036 |
| **Depression Symptomatology** | **1, 212** | **6.69** | **.010** |
| Anxiety Symptomatology | 1, 212 | 1.60 | .207 |
| Film Clip | 3, 636 | 1.69 | .170 |
| Film Clip X ΔRR | 3, 636 | 1.95 | .121 |
| Film Clip X Depression Symptomatology | 3, 636 | 0.93 | .426 |
| Film Clip X Anxiety Symptomatology | 3, 636 | 0.50 | .682 |
| Film Clip x Gender | 3, 636 | 1.05 | .368 |

Note: Bold lines indicate significant effects within the analyses reported in the main manuscript

1. **Sensitivity Analyses testing for the robustness of the current findings**

To test for the robustness of the current findings, a series of sensitivity analyses were run. We followed published recommendations and conducted Bootstrapping with 1000 samples (for details please consult Efron, 1987; Saltelli et al., 2004). These analyses were performed for *all* statistical tests reported in the original version of our manuscript. In fact, all results remained stable. Sections 4.1 to 4.x of this Supplementary Online Materials Files provide a detailed overview over the respective analyses concerning each of the statistical analyses performed in the main manuscript. All subsections are labeled accorinding to their appearance in the main manuscript.

**4.1. Are there diagnose-based differecnes in rRSA?**

One-way between subject ANOVA

Factor: HC, DEP, ANX

Anova Table (Type 3 tests)

| *Response: Value* | |  |  |  |  |  |  |  |  |  |
| --- | --- | --- | --- | --- | --- | --- | --- | --- | --- | --- |
|  | num Df | den Df | MSE | F | Pes | Pr(>F) | Mean effect size | Lower Limit effect size | Upper Limit effect size | Nbr. of tests |
| Factor1 | 2 | 226 | 2.17 | 13.04 | 0.1 | 0.07 | 0.11 | 0.06 | 0.17 | 1000 |

*Note*. MSE=Mean squared error,

| *Estimated Marginal Means* | | |  |  |  |  |  |
| --- | --- | --- | --- | --- | --- | --- | --- |
|  | Group | Est mean | Est CI Lower Limit | Est CI Upper Limit | SE mean | SE CI Lower Limit | SE CI Upper Limit |
| 1 | HC | 6.40 | 6.17 | 6.65 | 0.24 | 0.21 | 0.28 |
| 2 | DEP | 5.13 | 4.86 | 5.42 | 0.20 | 0.18 | 0.21 |
| 3 | ANX | 5.70 | 5.46 | 5.94 | 0.21 | 0.18 | 0.23 |

*Note*. Est=estimated, CI=confidence interval, SE=standard error

| *Post-hoc Tests* | |  |  |  |  |  |  |  |  |  |
| --- | --- | --- | --- | --- | --- | --- | --- | --- | --- | --- |
| *Number of Tests: 954* | | |  |  |  |  |  |  |  |  |
|  | Comparison | M_Diff_ | M_Diff_ CI Lower Limit | M_Diff_ CI Upper Limit | Est mean | Est CI Lower Limit | Est CI Upper Limit | SE mean | SE CI Lower Limit | SE CI Upper Limit |
| 1 | HC - DEP | 0.87 | 0.62 | 1.13 | 1.27 | 0.92 | 1.63 | 0.31 | 0.28 | 0.35 |
| 2 | HC - ANX | 0.48 | 0.26 | 0.72 | 0.71 | 0.37 | 1.04 | 0.32 | 0.29 | 0.35 |
| 3 | DEP - ANX | -0.39 | -0.65 | -0.13 | -0.57 | -0.90 | -0.20 | 0.28 | 0.26 | 0.31 |

*Note*. Est=estimated, CI=confidence interval, SE=standard error

**Summary:**

The bootstrapped results indicate that our findings are robust: F(2,226)=13.04 is significant at a typical alpha level (p < 0.05), indicating that the means of the groups (KG, Dep, Anx) differ. In Post Hoc tests, none of these confidence intervals contains zero, indicating significant group differences in all three comparisons***.***

**4.2. Are there transdiagnostic associations with rRSA**

| **Correlations** |  |  |  |  |  |  |  |
| --- | --- | --- | --- | --- | --- | --- | --- |
|  |  |  |  | rRSA | Depression | Angst | Stress |
| rRSA | Pearson Correlation | |  | - | -.246^**^ | -.207^**^ | -0.068 |
|  | Bootstrap^c^ | Bias |  | - | -0.003 | -0.001 | -0.004 |
|  |  | Std. Error |  | - | 0.064 | 0.064 | 0.065 |
|  |  | 95% CI | Lower | - | -0.373 | -0.330 | -0.208 |
|  |  |  | Upper | - | -0.124 | -0.079 | 0.056 |
| Depression | Pearson Correlation | |  | -.246^**^ | - | .596^**^ | .638^**^ |
|  | Bootstrap^c^ | Bias |  | -0.003 | - | -0.001 | 0.001 |
|  |  | Std. Error |  | 0.064 | - | 0.048 | 0.042 |
|  |  | 95% CI | Lower | -0.373 | - | 0.491 | 0.552 |
|  |  |  | Upper | -0.124 | - | 0.681 | 0.717 |
| Angst | Pearson Correlation | |  | -.207^**^ | .596^**^ | - | .573^**^ |
|  | Bootstrap^c^ | Bias |  | -0.001 | -0.001 | - | 0.004 |
|  |  | Std. Error |  | 0.064 | 0.048 | - | 0.049 |
|  |  | 95% CI | Lower | -0.330 | 0.491 | - | 0.473 |
|  |  |  | Upper | -0.079 | 0.681 | - | 0.668 |
| Stress | Pearson Correlation | |  | -0.068 | .638^**^ | .573^**^ | - |
|  | Bootstrap^c^ | Bias |  | -0.004 | 0.001 | 0.004 | - |
|  |  | Std. Error |  | 0.065 | 0.042 | 0.049 | - |
|  |  | 95% CI | Lower | -0.208 | 0.552 | 0.473 | - |
|  |  |  | Upper | 0.056 | 0.717 | 0.668 | - |
| **. Correlation is significant at the 0.01 level (2-tailed). | | | | |  |  |  |
| c. Unless otherwise noted, bootstrap results are based on 1000 bootstrap samples | | | | | | |  |

**Summary:**

The bootstrapped results indicate that rRSA significantly negative correlated with Depression and anxiety, but not stress.

**Linear Regression**

| *Coefficients^a^* |  |  |  |  |  |  |
| --- | --- | --- | --- | --- | --- | --- |
| Model | | Unstandardized Coefficients | | Standardized Coefficients | t | Sig. |
|  |  | B | Std. Error | Beta |  |  |
| 1 | (Constant) | 6.156 | 0.166 |  | 37.073 | 0.000 |
|  | Depression | -0.048 | 0.020 | -0.191 | -2.364 | 0.019 |
|  | Angst | -0.032 | 0.028 | -0.093 | -1.147 | 0.253 |
| a. Dependent Variable: LNHF_HRV_Base_welch60 | | | |  |  |  |

| *Bootstrap for Coefficients* | | | |  |  |  |  |
| --- | --- | --- | --- | --- | --- | --- | --- |
| Model | | B | Bootstrap^a^ | | | | |
|  |  |  | Bias | Std. Error | Sig. (2-tailed) | 95% Confidence Interval | |
|  |  |  |  |  |  | Lower | Upper |
| 1 | (Constant) | 6.156 | -0.002 | 0.163 | 0.001 | 5.837 | 6.475 |
|  | Depression | -0.048 | 0.000 | 0.021 | 0.022 | -0.090 | -0.007 |
|  | Angst | -0.032 | 0.001 | 0.030 | 0.282 | -0.091 | 0.025 |
| a. Unless otherwise noted. bootstrap results are based on 1000 bootstrap samples | | | | | | |  |

**Summary:**

The bootstrapped results indicate that the findings from linear regression are robust: Depression is a significant predictor (with negative coefficient) while anxiety not a significant predictor for rRSA.

**4.3. Do the film clips elicit significant RSA reactivity as compared to the resting baseline (ΔRSA)?**

One-way within subject ANOVA

Factor: Baseline, happy, neutral, threatening, sad film clip

Anova Table (Type 3 tests)

| *Response: Value* | |  |  |  |  |  |  |  |  |
| --- | --- | --- | --- | --- | --- | --- | --- | --- | --- |
|  | num Df | den Df | MSE | F | Pes | Pr(>F) | Mean  effect size | CI Lower Limit  effect size | CI Upper Limit  effect size |
| Factor1 | 3.68 | 805.7 | 0.35 | 14.06 | 0.06 | 0.58 | 0.06 | 0.04 | 0.1 |

*Note*. MSE=Mean squared error,

| *Estimated Marginal Means* | | | |  |  |  |  |
| --- | --- | --- | --- | --- | --- | --- | --- |
|  | Group | Est mean | Est mean CI  Lower Limit | Est mean CI  Upper Limit | SE mean | SE CI  Lower Limit | SE CI  Upper Limit |
| 1 | Baseline | 5.63 | 5.41 | 5.83 | 0.10 | 0.10 | 0.12 |
| 2 | Happy | 5.25 | 5.05 | 5.44 | 0.10 | 0.09 | 0.11 |
| 3 | Neutral | 5.30 | 5.11 | 5.50 | 0.10 | 0.10 | 0.11 |
| 4 | Threatening | 5.38 | 5.19 | 5.56 | 0.10 | 0.09 | 0.11 |
| 5 | Sad | 5.41 | 5.24 | 5.60 | 0.09 | 0.09 | 0.10 |

*Note*. Est=estimated, CI=confidence interval, SE=standard error

| *Post-hoc Tests* | | | | | |  |  |  |  |  |  |
| --- | --- | --- | --- | --- | --- | --- | --- | --- | --- | --- | --- |
| *Number of Tests: 998* | | | | | |  |  |  |  |  |  |
|  | | Comparison | M_Diff_ | M_Diff_ CI  Lower Limit | M_diff_ CI  Upper Limit | Est mean | Est CI  Lower Limit | Est CI  Upper Limit | SE mean | SE CI Lower Limit | SE CI Upper Limit |
| 1 | Baseline - Happy | | 0.11 | 0.08 | 0.15 | 0.37 | 0.26 | 0.49 | 0.06 | 0.05 | 0.07 |
| 2 | Baseline - Neutral | | 0.10 | 0.06 | 0.13 | 0.32 | 0.22 | 0.42 | 0.05 | 0.05 | 0.06 |
| 3 | Baseline - Threatening | | 0.07 | 0.04 | 0.11 | 0.25 | 0.14 | 0.36 | 0.06 | 0.05 | 0.07 |
| 4 | Baseline - Sad | | 0.06 | 0.03 | 0.10 | 0.21 | 0.10 | 0.32 | 0.06 | 0.05 | 0.07 |
| 5 | Happy - Neutral | | -0.02 | -0.05 | 0.01 | -0.05 | -0.16 | 0.05 | 0.05 | 0.05 | 0.06 |
| 6 | Happy - Threatening | | -0.04 | -0.06 | -0.01 | -0.13 | -0.21 | -0.04 | 0.05 | 0.04 | 0.05 |
| 7 | Happy - Sad | | -0.05 | -0.08 | -0.02 | -0.16 | -0.26 | -0.06 | 0.05 | 0.05 | 0.06 |
| 8 | Neutral - Threatening | | -0.02 | -0.05 | 0.01 | -0.07 | -0.17 | 0.03 | 0.05 | 0.04 | 0.06 |
| 9 | Neutral - Sad | | -0.03 | -0.07 | 0.00 | -0.11 | -0.22 | 0.00 | 0.06 | 0.05 | 0.06 |
| 10 | Threatening - Sad | | -0.01 | -0.04 | 0.02 | -0.04 | -0.13 | 0.05 | 0.05 | 0.04 | 0.05 |

*Note*. Est=estimated, CI=confidence interval, SE=standard error

**Summary:**

The bootstrapped results indicate that RSA significantly differed within the four film clips F(3.68, 805.7)=14.06 (p<0.05). ). In Post hoc tests: the confidence intervals of baseline vs happy, baseline vs neutral, baseline vs fear and baseline vs sad contain no zeros, which indicates significant differences.

**4.4. Are there diagnose-based difference in ΔRSA?**

Two-way mixed ANOVA

Factor 1: Gruppe (HC, DEP, ANX)

Factor 2: Film Clip (happy, neutral, Threatening, sad)

Anova Table (Type 3 tests)

| *Response: Value* | | |  |  |  |  |  |  |  |  |
| --- | --- | --- | --- | --- | --- | --- | --- | --- | --- | --- |
|  | num Df | den Df | MSE | F | Pes | Pr(>F) | Mean effect size | Lower Limit effect size | Upper Limit effect size | Nbr. of tests |
| Factor1 | 2.00 | 217.00 | 2.17 | 1.29 | 0.01 | 0.65 | 0.02 | 0.00 | 0.06 | 1000 |
| Factor2 | 2.86 | 619.87 | 0.23 | 5.76 | 0.03 | 0.59 | 0.03 | 0.01 | 0.06 | 1000 |
| Factor1:Factor2 | 5.71 | 619.87 | 0.23 | 0.54 | 0.00 | 0.89 | 0.01 | 0.00 | 0.03 | 1000 |

*Note*. MSE=Mean squared error,

| *Estimated Marginal Means Factor 1* | | |  |  |  |  |  |
| --- | --- | --- | --- | --- | --- | --- | --- |
|  | Group | Est mean | Est CI Lower Limit | Est CI Upper Limit | SE mean | SE CI Lower Limit | SE CI Upper Limit |
| 1 | HC | -0.27 | -0.47 | -0.08 | 0.10 | 0.08 | 0.12 |
| 2 | DEP | -0.19 | -0.34 | -0.04 | 0.08 | 0.07 | 0.09 |
| 3 | ANX | -0.38 | -0.56 | -0.23 | 0.08 | 0.07 | 0.10 |

*Note*. Est=estimated, CI=confidence interval, SE=standard error

| *Post-hoc Tests Factor 1* | | | | |  |  |  |  |  |  |
| --- | --- | --- | --- | --- | --- | --- | --- | --- | --- | --- |
| *Number of Tests: 971* | | | | |  |  |  |  |  |  |
|  | Comparison | M_Diff_ | M_Diff_ CI Lower Limit | M_Diff_ CI Upper Limit | Est mean | Est CI Lower Limit | Est CI Upper Limit | SE mean | SE CI Lower Limit | SE CI Upper Limit |
| 1 | HC - DEP | -0.05 | -0.20 | 0.11 | -0.08 | -0.34 | 0.18 | 0.13 | 0.11 | 0.15 |
| 2 | HC - ANX | 0.06 | -0.09 | 0.22 | 0.11 | -0.15 | 0.38 | 0.13 | 0.11 | 0.15 |
| 3 | DEP - ANX | 0.11 | -0.03 | 0.24 | 0.19 | -0.04 | 0.42 | 0.11 | 0.10 | 0.13 |

*Note*. Est=estimated, CI=confidence interval, SE=standard error

| *Estimated Marginal Means Factor 2* | | |  |  |  |  |  |
| --- | --- | --- | --- | --- | --- | --- | --- |
|  | Group | Est mean | Est CI Lower Limit | Est CI Upper Limit | SE mean | SE CI Lower Limit | SE CI Upper Limit |
| 1 | Happy | -0.37 | -0.49 | -0.26 | 0.06 | 0.05 | 0.07 |
| 2 | Neutral | -0.32 | -0.42 | -0.21 | 0.05 | 0.05 | 0.06 |
| 3 | Threatening | -0.23 | -0.35 | -0.12 | 0.06 | 0.05 | 0.07 |
| 4 | Sad | -0.20 | -0.31 | -0.10 | 0.06 | 0.05 | 0.06 |

*Note*. Est=estimated, CI=confidence interval, SE=standard error

| *Post-hoc Tests Factor 2* | | | | | |  |  |  |  |  |
| --- | --- | --- | --- | --- | --- | --- | --- | --- | --- | --- |
| *Number of Tests: 999* | | | | | |  |  |  |  |  |
|  | Comparison | M_diff_ | M_diff_ CI Lower Limit | M_Diff_ CI Upper Limit | Est mean | Est CI Lower Limit | Est CI Upper Limit | SE mean | SE CI Lower Limit | SE CI Upper Limit |
| 1 | Happy - Neutral | -0.03 | -0.09 | 0.02 | -0.06 | -0.15 | 0.03 | 0.05 | 0.04 | 0.06 |
| 2 | Happy - Threatening | -0.08 | -0.14 | -0.03 | -0.14 | -0.22 | -0.06 | 0.04 | 0.03 | 0.05 |
| 3 | Happy - Sad | -0.10 | -0.15 | -0.05 | -0.17 | -0.25 | -0.09 | 0.05 | 0.04 | 0.05 |
| 4 | Neutral - Threatening | -0.05 | -0.11 | 0.01 | -0.08 | -0.18 | 0.01 | 0.05 | 0.04 | 0.06 |
| 5 | Neutral - Sad | -0.07 | -0.12 | -0.01 | -0.11 | -0.21 | -0.03 | 0.05 | 0.04 | 0.06 |
| 6 | Threatening - Sad | -0.02 | -0.06 | 0.03 | -0.03 | -0.10 | 0.04 | 0.04 | 0.03 | 0.05 |

*Note*. Est=estimated, CI=confidence interval, SE=standard error

| *Estimated Marginal Means Factor 1 by Factor 2* | | | | | |  |  |  |
| --- | --- | --- | --- | --- | --- | --- | --- | --- |
|  | Group factor 2 | Group factor 1 | Est mean | Est CI Lower Limit | Est CI Upper Limit | SE mean | SE CI Lower Limit | SE CI Upper Limit |
| 1 | Happy | HC | -0.33 | -0.54 | -0.12 | 0.12 | 0.10 | 0.15 |
| 2 | Happy | DEP | -0.27 | -0.45 | -0.08 | 0.10 | 0.08 | 0.11 |
| 3 | Happy | ANX | -0.52 | -0.75 | -0.32 | 0.10 | 0.08 | 0.12 |
| 4 | Neutral | HC | -0.33 | -0.56 | -0.09 | 0.11 | 0.09 | 0.12 |
| 5 | Neutral | DEP | -0.26 | -0.41 | -0.10 | 0.08 | 0.07 | 0.10 |
| 6 | Neutral | ANX | -0.36 | -0.51 | -0.19 | 0.09 | 0.08 | 0.10 |
| 7 | Threatening | HC | -0.22 | -0.45 | 0.02 | 0.11 | 0.09 | 0.15 |
| 8 | Threatening | DEP | -0.13 | -0.27 | 0.02 | 0.09 | 0.07 | 0.11 |
| 9 | Threatening | ANX | -0.35 | -0.61 | -0.15 | 0.10 | 0.08 | 0.12 |
| 10 | Sad | HC | -0.20 | -0.39 | 0.00 | 0.11 | 0.09 | 0.13 |
| 11 | Sad | DEP | -0.12 | -0.30 | 0.06 | 0.09 | 0.08 | 0.10 |
| 12 | Sad | ANX | -0.29 | -0.49 | -0.13 | 0.09 | 0.08 | 0.11 |

*Note*. Est=estimated, CI=confidence interval, SE=standard error, HC=healthy control, DEP=Depression, ANX=anxiety

| *Post-hoc Tests Factor 1 by Factor 2* | | | | | | | |  |  |  |  |
| --- | --- | --- | --- | --- | --- | --- | --- | --- | --- | --- | --- |
| *Number of Tests: 971* | | | | | | | |  |  |  |  |
|  | Group factor 2 | Comparison | M_diff_ | M_Diff_ CI Lower Limit | M_Diff_ CI Upper Limit | Est mean | Est CI Lower Limit | Est CI Upper Limit | SE mean | SE CI Lower Limit | SE CI Upper Limit |
| 1 | Happy | HC - DEP | -0.04 | -0.22 | 0.13 | -0.07 | -0.36 | 0.21 | 0.15 | 0.13 | 0.18 |
| 2 | Happy | HC - ANX | 0.11 | -0.07 | 0.28 | 0.19 | -0.11 | 0.50 | 0.16 | 0.13 | 0.19 |
| 3 | Happy | DEP - ANX | 0.15 | -0.03 | 0.31 | 0.25 | -0.04 | 0.54 | 0.14 | 0.12 | 0.16 |
| 4 | Neutral | HC - DEP | -0.04 | -0.21 | 0.13 | -0.07 | -0.35 | 0.22 | 0.14 | 0.12 | 0.15 |
| 5 | Neutral | HC - ANX | 0.01 | -0.16 | 0.19 | 0.02 | -0.26 | 0.31 | 0.14 | 0.13 | 0.15 |
| 6 | Neutral | DEP - ANX | 0.06 | -0.08 | 0.20 | 0.10 | -0.14 | 0.33 | 0.12 | 0.11 | 0.14 |
| 7 | Threatening | HC - DEP | -0.06 | -0.23 | 0.10 | -0.09 | -0.37 | 0.17 | 0.15 | 0.12 | 0.18 |
| 8 | Threatening | HC - ANX | 0.08 | -0.12 | 0.27 | 0.13 | -0.19 | 0.47 | 0.15 | 0.12 | 0.19 |
| 9 | Threatening | DEP - ANX | 0.13 | -0.02 | 0.28 | 0.23 | -0.03 | 0.50 | 0.13 | 0.11 | 0.16 |
| 10 | Sad | HC - DEP | -0.04 | -0.21 | 0.12 | -0.07 | -0.35 | 0.20 | 0.14 | 0.12 | 0.16 |
| 11 | Sad | HC - ANX | 0.06 | -0.12 | 0.22 | 0.10 | -0.18 | 0.37 | 0.14 | 0.13 | 0.17 |
| 12 | Sad | DEP - ANX | 0.10 | -0.05 | 0.25 | 0.17 | -0.08 | 0.43 | 0.13 | 0.11 | 0.14 |

*Note*. Est=estimated, CI=confidence interval, SE=standard error, HC=healthy control, DEP=Depression, ANX=anxiety

| *Estimated Marginal Means Factor 2 by Factor 1* | | | | | | |  |  |
| --- | --- | --- | --- | --- | --- | --- | --- | --- |
|  | Group factor 1 | Group factor 2 | Est mean | Est CI Lower Limit | Est CI Upper Limit | SE mean | SE CI Lower Limit | SE CI Upper Limit |
| 1 | HC | Happy | -0.33 | -0.54 | -0.12 | 0.12 | 0.10 | 0.15 |
| 2 | HC | Neutral | -0.33 | -0.57 | -0.10 | 0.11 | 0.09 | 0.12 |
| 3 | HC | Threatening | -0.22 | -0.45 | 0.02 | 0.11 | 0.09 | 0.15 |
| 4 | HC | Sad | -0.20 | -0.39 | 0.00 | 0.11 | 0.09 | 0.13 |
| 5 | DEP | Happy | -0.27 | -0.45 | -0.08 | 0.10 | 0.08 | 0.11 |
| 6 | DEP | Neutral | -0.26 | -0.41 | -0.10 | 0.08 | 0.07 | 0.09 |
| 7 | DEP | Threatening | -0.13 | -0.27 | 0.02 | 0.09 | 0.07 | 0.11 |
| 8 | DEP | Sad | -0.12 | -0.31 | 0.06 | 0.09 | 0.08 | 0.10 |
| 9 | ANX | Happy | -0.52 | -0.75 | -0.32 | 0.10 | 0.08 | 0.12 |
| 10 | ANX | Neutral | -0.36 | -0.51 | -0.19 | 0.09 | 0.08 | 0.10 |
| 11 | ANX | Threatening | -0.35 | -0.60 | -0.15 | 0.09 | 0.08 | 0.12 |
| 12 | ANX | Sad | -0.29 | -0.48 | -0.13 | 0.09 | 0.08 | 0.11 |

*Note*. Est=estimated, CI=confidence interval, SE=standard error, HC=healthy control, DEP=Depression, ANX=anxiety

| *Post-hoc Tests Factor 2 by Factor 1* | | | | | | | |  |  |  |  |
| --- | --- | --- | --- | --- | --- | --- | --- | --- | --- | --- | --- |
| *Number of Tests: 1000* | | | | | | | |  |  |  |  |
|  | Group factor 1 | Comparison | M_Diff_ | M_Diff_ CI Lower Limit | M_Diff_ CI Upper Limit | Est mean | Est CI Lower Limit | Est CI Upper Limit | SE mean | SE CI Lower Limit | SE CI Upper Limit |
| 1 | HC | Happy - Neutral | 0.00 | -0.09 | 0.08 | 0.00 | -0.14 | 0.13 | 0.10 | 0.08 | 0.12 |
| 2 | HC | Happy - Threatening | -0.07 | -0.17 | 0.02 | -0.11 | -0.28 | 0.04 | 0.08 | 0.07 | 0.10 |
| 3 | HC | Happy - Sad | -0.08 | -0.16 | -0.01 | -0.14 | -0.25 | -0.01 | 0.09 | 0.08 | 0.11 |
| 4 | HC | Neutral - Threatening | -0.07 | -0.17 | 0.03 | -0.11 | -0.29 | 0.06 | 0.09 | 0.07 | 0.11 |
| 5 | HC | Neutral - Sad | -0.08 | -0.17 | 0.01 | -0.13 | -0.28 | 0.02 | 0.10 | 0.08 | 0.12 |
| 6 | HC | Threatening - Sad | -0.01 | -0.09 | 0.07 | -0.02 | -0.14 | 0.11 | 0.08 | 0.06 | 0.09 |
| 7 | DEP | Happy - Neutral | 0.00 | -0.09 | 0.08 | -0.01 | -0.15 | 0.13 | 0.08 | 0.06 | 0.09 |
| 8 | DEP | Happy - Threatening | -0.09 | -0.16 | -0.01 | -0.14 | -0.26 | -0.02 | 0.06 | 0.05 | 0.08 |
| 9 | DEP | Happy - Sad | -0.09 | -0.18 | 0.01 | -0.15 | -0.30 | 0.02 | 0.07 | 0.06 | 0.08 |
| 10 | DEP | Neutral - Threatening | -0.08 | -0.14 | -0.02 | -0.13 | -0.23 | -0.03 | 0.07 | 0.06 | 0.09 |
| 11 | DEP | Neutral - Sad | -0.08 | -0.17 | 0.00 | -0.14 | -0.28 | 0.00 | 0.08 | 0.07 | 0.09 |
| 12 | DEP | Threatening - Sad | 0.00 | -0.07 | 0.06 | 0.00 | -0.11 | 0.11 | 0.06 | 0.05 | 0.07 |
| 13 | ANX | Happy - Neutral | -0.10 | -0.21 | 0.01 | -0.16 | -0.37 | 0.02 | 0.08 | 0.07 | 0.10 |
| 14 | ANX | Happy - Threatening | -0.10 | -0.19 | -0.02 | -0.17 | -0.31 | -0.03 | 0.07 | 0.06 | 0.08 |
| 15 | ANX | Happy - Sad | -0.13 | -0.23 | -0.03 | -0.22 | -0.39 | -0.05 | 0.08 | 0.07 | 0.09 |
| 16 | ANX | Neutral - Threatening | 0.00 | -0.11 | 0.11 | 0.00 | -0.17 | 0.20 | 0.07 | 0.06 | 0.09 |
| 17 | ANX | Neutral - Sad | -0.04 | -0.14 | 0.06 | -0.06 | -0.23 | 0.11 | 0.08 | 0.07 | 0.09 |
| 18 | ANX | Threatening - Sad | -0.03 | -0.12 | 0.05 | -0.06 | -0.21 | 0.09 | 0.06 | 0.05 | 0.08 |

*Note*. Est=estimated, CI=confidence interval, SE=standard error, HC=healthy control, DEP=Depression, ANX=anxiety

**Summary:**

Bootstrap results indicate the findings are robust: ΔRSA differed significantly within the four film clips F(2.86, 619.87)=5.76 (p<.05). In Post hoc tests: the confidence intervals of happy vs Threaten, happy vs sad, neutral vs sad contain no zeros, which indicates significant differences. F(2, 217)=1.29 and F(5.71, 619.87)=0.54 indicate neither significant between factor nor interaction.

**4.5.** **Are there transdiagnostic associations with ΔRSA**

| *Bootstrap Specifications* | | | |  |  |  |  |  |
| --- | --- | --- | --- | --- | --- | --- | --- | --- |
| Sampling Method | | Simple | |  |  |  |  |  |
| Number of Samples | | 1000 | |  |  |  |  |  |
| Confidence Interval Level | | 95.0% | |  |  |  |  |  |
| Confidence Interval Type | | Percentile | |  |  |  |  |  |
| *Multivariate Tests^a^* | | | | | | | | |
| Effect | | | Value | | F | Hypothesis df | Error df | Sig. |
| Intercept | Pillai's Trace | | .069 | | 3.940^b^ | 4.000 | 211.000 | .004 |
|  | Wilks' Lambda | | .931 | | 3.940^b^ | 4.000 | 211.000 | .004 |
|  | Hotelling's Trace | | .075 | | 3.940^b^ | 4.000 | 211.000 | .004 |
|  | Roy's Largest Root | | .075 | | 3.940^b^ | 4.000 | 211.000 | .004 |
| DASS_Depr_pre | Pillai's Trace | | .048 | | 2.667^b^ | 4.000 | 211.000 | .033 |
|  | Wilks' Lambda | | .952 | | 2.667^b^ | 4.000 | 211.000 | .033 |
|  | Hotelling's Trace | | .051 | | 2.667^b^ | 4.000 | 211.000 | .033 |
|  | Roy's Largest Root | | .051 | | 2.667^b^ | 4.000 | 211.000 | .033 |
| DASS_Angst_pre | Pillai's Trace | | .014 | | .728^b^ | 4.000 | 211.000 | .574 |
|  | Wilks' Lambda | | .986 | | .728^b^ | 4.000 | 211.000 | .574 |
|  | Hotelling's Trace | | .014 | | .728^b^ | 4.000 | 211.000 | .574 |
|  | Roy's Largest Root | | .014 | | .728^b^ | 4.000 | 211.000 | .574 |
| DASS_Stress_pre | Pillai's Trace | | .029 | | 1.599^b^ | 4.000 | 211.000 | .176 |
|  | Wilks' Lambda | | .971 | | 1.599^b^ | 4.000 | 211.000 | .176 |
|  | Hotelling's Trace | | .030 | | 1.599^b^ | 4.000 | 211.000 | .176 |
|  | Roy's Largest Root | | .030 | | 1.599^b^ | 4.000 | 211.000 | .176 |
| *Note*. a. Design: Intercept + DASS_Depr_pre + DASS_Angst_pre + DASS_Stress_pre | | | | | | | | |
| b. Exact statistic | | | | | | | | |

**Summary**

The bootstrapped results indicate that our findings are robust: only depression is a significant predictor for ΔRSA (with negative coefficients).

**4.6. Prediction of treatment Outcome with markers of RSA**

| Pearson Correlation | | | Post Treatment Global Success Rating | | DASS General Distress Residual Gain Score | |
| --- | --- | --- | --- | --- | --- | --- |
|  |  |  | Depression | Anxiety | Depression | Anxiety |
| rRSA |  |  | 0.087 | 0.173 | -0.074 | -0.103 |
| Bootstrapc | Bias |  | 0.001 | 0.001 | 0 | -0.008 |
|  | Std. Error |  | 0.116 | 0.139 | 0.125 | 0.163 |
|  | 95% Confidence Interval | Lower | -0.138 | -0.099 | -0.313 | -0.458 |
|  |  | Upper | 0.32 | 0.446 | 0.173 | 0.186 |
| ΔRSA _hap_ |  |  | -.320** | -0.2 | .299* | 0.096 |
| Bootstrapc | Bias |  | 0.011 | 0.003 | -0.004 | -0.002 |
|  | Std. Error |  | 0.136 | 0.147 | 0.111 | 0.117 |
|  | 95% Confidence Interval | Lower | -0.545 | -0.474 | 0.059 | -0.143 |
|  |  | Upper | -0.01 | 0.098 | 0.514 | 0.313 |
| ΔRSA _neut_ |  |  | -.252* | 0.157 | .275* | -0.079 |
| Bootstrapc | Bias |  | 0.004 | -0.005 | -0.004 | -0.004 |
|  | Std. Error |  | 0.141 | 0.164 | 0.107 | 0.142 |
|  | 95% Confidence Interval | Lower | -0.502 | -0.18 | 0.049 | -0.348 |
|  |  | Upper | 0.05 | 0.454 | 0.471 | 0.197 |
| ΔRSA _threat_ |  |  | -.310* | 0.065 | .291* | -0.092 |
| Bootstrapc | Bias |  | 0.006 | 0.006 | -0.003 | -0.005 |
|  | Std. Error |  | 0.139 | 0.144 | 0.094 | 0.124 |
|  | 95% Confidence Interval | Lower | -0.559 | -0.185 | 0.101 | -0.352 |
|  |  | Upper | -0.006 | 0.367 | 0.47 | 0.14 |
| ΔRSA _sad_ |  |  | -.262* | 0.115 | .262* | -0.067 |
| Bootstrapc | Bias |  | 0.003 | 0 | -0.001 | -0.006 |
|  | Std. Error |  | 0.125 | 0.171 | 0.088 | 0.149 |
|  | 95% Confidence Interval | Lower | -0.491 | -0.231 | 0.086 | -0.358 |
|  |  | Upper | 0.003 | 0.445 | 0.43 | 0.227 |

*Note*. Hap=happy film clip, neut=neutral film clip, threat=threatening film clip, sad=sad film clip Depression n=67, anxiety n=49. Significance = * p <0,05, ** p <0,01, *** p <0,001.

**Summary:**

The bootstrapped results indicate that our findings are robust: rRSA was not significantly associated with the two treatment outcome measures, neither for depressed, nor for patients with anxiety disorders. ΔRSA towards all four films was associated with better treatment outcome on both outcome measures only in the depression group.
